# Source and fractionation controls on subduction-related plutons and dike swarms in southern Patagonia (Torres del Paine area) and the low Nb/Ta of upper crustal igneous rocks

**DOI:** 10.1007/s00410-018-1467-0

**Published:** 2018-04-19

**Authors:** Othmar Müntener, Tanya Ewing, Lukas P. Baumgartner, Mélina Manzini, Thibaud Roux, Pierre Pellaud, Luc Allemann

**Affiliations:** 1 Institute of Earth Sciences, University of Lausanne, Géopolis, 1015 Lausanne, Switzerland; 2 Present Address: Institute of Geological Sciences, University of Bern, Baltzerstrasse 1+3, 3012 Bern, Switzerland

**Keywords:** Calc-alkaline magmatism, Arc migration, Nb/Ta ratio, Torres del Paine, Zircon, Geochronology

## Abstract

The subduction system in southern Patagonia provides direct evidence for the variability of the position of an active continental arc with respect to the subducting plate through time, but the consequences on the arc magmatic record are less well studied. Here we present a geochemical and geochronological study on small plutons and dykes from the upper crust of the southern Patagonian Andes at ~ 51°S, which formed as a result of the subduction of the Nazca and Antarctic plates beneath the South American continent. In situ U–Pb geochronology on zircons and bulk rock geochemical data of plutonic and dyke rocks are used to constrain the magmatic evolution of the retro-arc over the last 30 Ma. We demonstrate that these combined U–Pb and geochemical data for magmatic rocks track the temporal and spatial migration of the active arc, and associated retro-arc magmatism. Our dataset indicates that the rear-arc area is characterized by small volumes of alkaline basaltic magmas at 29–30 Ma that are characterized by low La/Nb and Th/Nb ratios with negligible arc signatures. Subsequent progressive eastward migration of the active arc culminated with the emplacement of calc-alkaline plutons and dikes ~ 17–16 Ma with elevated La/Nb and Th/Nb ratios and typical subduction signatures constraining the easternmost position of the southern Patagonian batholith at that time. Geochemical data on the post-16 Ma igneous rocks including the Torres del Paine laccolith indicate an evolution to transitional K-rich calc-alkaline magmatism at 12.5 ± 0.2 Ma. We show that trace element ratios such as Nb/Ta and Dy/Yb systematically decrease with increasing SiO_2_, for both the 17–16 Ma calc-alkaline and the 12–13 Ma K-rich transitional magmatism. In contrast, Th/Nb and La/Nb monitor the changes in the source composition of these magmas. We suggest that the transition from the common calc-alkaline to K-rich transitional magmatism involves a change in the source component, while the trace element ratios, such as Nb/Ta and Dy/Yb, of derivative higher silica content liquids are controlled by similar fractionating mineral assemblages. Analysis of a global compilation of Nb/Ta ratios of arc magmatic rocks and simple geochemical models indicate that amphibole and variable amounts of biotite exert a major control on the low Dy/Yb and Nb/Ta of derivative granitic liquids. Lastly, we suggest that the low Nb/Ta ratio of silica-rich magmas is a natural consequence of biotite fractionation and that alternative models such as amphibolite melting in subduction zones and diffusive fractionation are not required to explain the Nb/Ta ratio of the upper continental crust.

**Electronic supplementary material:**

The online version of this article (10.1007/s00410-018-1467-0) contains supplementary material, which is available to authorized users.

## Introduction

Differentiation of the crust–mantle system in arcs is a fundamental process to understand the composition and the chemical evolution of the continental crust. Significant geochemical variation of arc magmatic products over time is the combined result of mantle melting, slab fluxes and crustal differentiation processes, which in turn depend on ‘subduction parameters’ such as convergence rate, dip angle of the subducting plate, the thermal structure of the mantle wedge and nature of the overriding plate. Temporally resolved geochemical data on arc magmatic products thus provide an opportunity to better characterize the dynamic evolution of a subduction system. While the nature of the slab contribution has been extensively studied by means of major, trace element, and isotope geochemistry of individual active volcanoes or entire arc segments, studies addressing the beginnings and endings of plutonic arc magmatic products in the same area have received less attention. Understanding why primitive arc magmatic products change over time and how they differentiate to silica-rich compositions is essential to understand the growth of the continental crust via magmatic additions. There is a consensus that amphibole has a prominent role in controlling some geochemical signatures of granitic rocks (e.g. Frey et al. 1978) and of the continental crust (e.g. Cawthorn et al. 1973; Davidson et al. 2007; Dessimoz et al. 2012; Blatter et al. 2013, 2017; Nandedkar et al. 2014). Specifically, amphibole is required to produce significant volumes of silica-rich arc magmas (e.g. Cawthorn and Brown 1976; Jagoutz et al. 2011). One of the more controversial issues is to what extent amphibole controls the evolution of some trace elements during differentiation, in particular rare earth elements (REE) and high-field strength elements, such as Ti, Nb and Ta. Several hypotheses have been advanced to explain the low Nb/Ta ratio of the continental crust, such as amphibolite melting in subduction zones (Foley et al. 2002), refractory rutile-bearing eclogites in the mantle (Rudnick et al. 2000), and granite formation by partial melting of lower crustal rocks (Stepanov et al. 2014). Most studies focused on Fe–Ti oxides as Nb–Ta are moderately to strongly compatible in rutile, ilmenite, titanite and Ti–magnetite (Green and Pearson 1986; Schmidt et al. 2004; Prowatke and Klemme 2005; Xiong et al. 2011). Yet experimental data have shown that liquids in equilibrium with rutile, ilmenite or titanite have superchondritic Nb/Ta ratios (e.g. Tiepolo et al. 2002; Schmidt et al. 2004) and, therefore, melting of eclogite or any other rock type with residual Fe–Ti oxides cannot explain the subchondritic Nb/Ta ratio of the upper continental crust. This has inspired disequilibrium models such as kinetic fractionation of Nb and Ta during partial melting (Marschall et al. 2013), yet the simplest model—fractionation of major Ti-bearing silicates that are able to fractionate high-field strength elements in shallow magmatic reservoirs—has received comparably little attention.

Here we focus on constraints from southern Patagonia, as an example to illustrate the governing processes. The main contribution from this paper is to show that (a) the geochemistry of plutons and dikes from the Torres del Paine area have changed significantly over the last 30 Ma, (b) this time-resolved geochemical dataset indicates that the Torres del Paine area was situated in back-arc position at ~ 30my and today, but represented the easternmost part of the magmatic arc between ~ 17 and 12 my, and (c) amphibole + biotite control some critical trace element ratios during fractionation from intermediate to high-Si magmas.

The approach of using detailed geochemical investigations of plutonic rocks and dikes to constrain the arc magmatic history is inspired from observations of modern subduction zone volcanoes. A number of studies addressed compositional variability over short timescales (< 10^5^ years) in single subduction zones (e.g. Tatsumi et al. 1983; Grove et al. 2002). Rapidly changing convergence rates might involve changes of the subducting angle and/or changes of subduction erosion rates of the forearc (Kay et al. 2005; Thomson et al. 2001; Ramírez de Arellano et al. 2012), which influence the composition of the subducting material, and consequently the slab component of subduction-related magmatism. The Cascade arc provides an example that is characterized by changing magmatic products, from H_2_O-poor olivine tholeiitic to H_2_O-rich calc-alkaline magmatism (e.g. DuBray et al. 2006), over timescales of less than 1 Ma (e.g. Grove et al. 2002). Such abrupt changes in volcanic systems are understood by an interference of decompression and flux melting in the mantle wedge and varying addition of a slab component to an active volcanic system over short timescales (Grove et al. 2002). Here we expand these concepts to an arc plutonic system that has assembled a series of plutons and dikes swarms over a well-constrained area to investigate the geochemical variability as a function of time. We present geochemical data from the Torres del Paine area, which shows two different generations of alkaline magmas, as well as two distinct calc-alkaline fractionation sequences. Combined with LA-ICPMS U–Pb dating on zircon we demonstrate that the geochemical record as preserved in plutons and dikes provide robust constraints on the effects of the migrating influence of arc magmatism on the magmatic record in the Torres del Paine area ranging from 30 Ma to the present.

### Large-scale geological setting

**Fig. 1:**
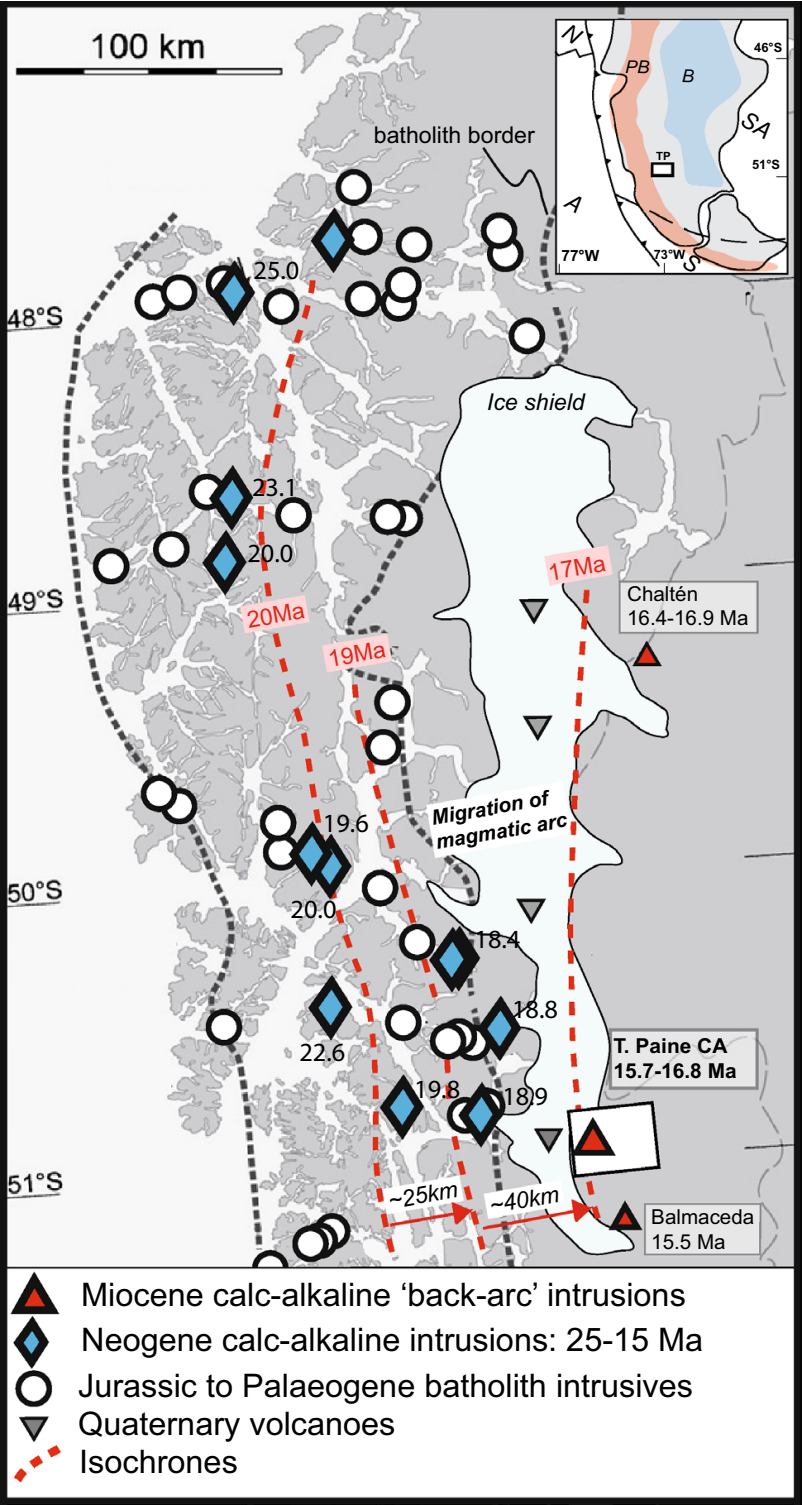
Simplified sketch map featuring dated plutons of the Patagonian batholith between 48 and 51°S, modified from Ramírez de Arellano et al. (2012) and the basic map of Hervé et al. (2007). Age of Balmaceda from Sanchez (2011). Inset shows geographical overview of Southern South America. *A* Antarctic plate, *N* Nazca plate, *SA* South American plate, *S* Scotia plate, *PB* Patagonian batholith, *B* plateau basalts

The studied area at 51°S is part of the Andean cordillera in Southern Patagonia, which is characterized by three major lithotectonic units (inset of Fig. 1). The ~ 300-km-long South Patagonian Batholith is the product of subduction of the Nazca and Antarctic plates beneath the South American plate from the Late Jurassic to the present (e.g. Hervé et al. 2007). It is emplaced into predominantly Paleozoic and Mesozoic metasedimentary sequences. These sequences are overlain by the late Jurassic Chon Aike magmatic province, which formed in response to extension related to the breakup of Gondwana (e.g. Pankhurst et al. 1999). Throughout the Cretaceous, sedimentary systems provided batholith-sourced material to the Andean foreland basin to the East of the Cordillera. In the late Cretaceous, the study area was affected by a compressional evolution that uplifted the Cretaceous basin infill, leading to the Patagonian fold and thrust belt (e.g. Ramos 1989; Fildani and Hessler 2005; Fosdick et al. 2011). Tertiary foreland basin infills to the East are separated by a major Paleocene unconformity from the underlying fold and thrust belt (e.g. Malumián et al. 1999). To the east of the batholith there are large areas with alkaline to transitional alkaline plateau lavas that formed from the late Cretaceous to the Tertiary and locally into the Holocene (e.g. Tyrrell 1932; Stern et al. 1990; Ramos et al. 2004). In the following, we summarize the most recent (~ 50 Ma) tectonic evolution of southern Patagonia relevant to our study.

Convergence rates and obliquity of subduction varied considerably through time, with highly oblique convergence from at least 42 Ma until ~ 25 Ma (Cande and Leslie 1986). Anomalously fast and nearly orthogonal convergence started at ~ 26 Ma, and slowed down at ~ 16 Ma (Cande and Leslie 1986; Somoza 1998; Breitsprecher and Thorkelson 2009). Beginning at ~ 14 Ma, the active spreading ridge between the Nazca and Antarctic plates (Chile ridge) arrived at the convergent margin in southernmost Patagonia (~ 53°S) and began to subduct (Cande and Leslie 1986; Breitsprecher and Thorkelson 2009). The associated triple junction episodically migrated north to reach its present position at ~ 46°S (Cande and Leslie 1986).

**Fig. 2:**
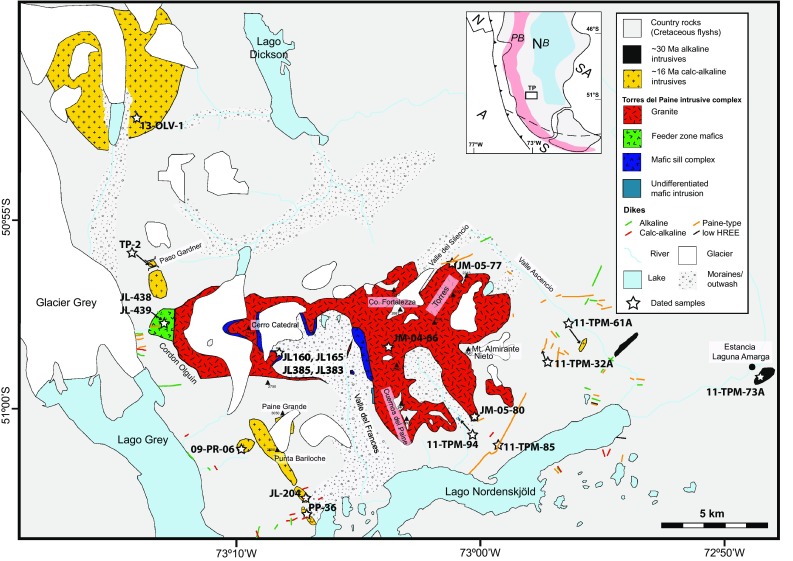
Simplified geological map of the Torres del Paine (TP) area with location of the different plutons and dikes. Dated samples are given with full sample number. Dikes within the felsic and mafic rocks of the Torres del Paine are not shown. Inset shows geographical overview of Southern South America as in Fig. 1. Sample names for the dikes can be found in Figure S1 and Table S1

It has been proposed that arc magmatism repeatedly shifted to the East over the last 100 Ma (Folguera and Ramos 2011). Of particular interest is the igneous evolution during the last 30 Ma. South of the present-day triple junction, this was characterized by eastward arc migration between ~ 20 and 16 Ma (Fig. 1). Based on a compilation of existing age data, it was proposed that the active arc as defined by ages of Patagonian batholith plutonic rocks migrated about 100 km to the east between ~ 20 and ~ 16 my (see Fig. 8 in Ramírez de Arellano et al. 2012). It was argued to give rise to calc-alkaline magmatism in the Fitz Roy–Cerro Torre area. The active volcanoes of the Southern Andes also testify to this shift in the locus of arc magmatism: south of 46°S all Quaternary volcanoes are found 40–80 km inboard of the exposed Patagonian batholith, whereas north of the triple junction modern volcanism occurs in the same geographic position as the batholith (Stern and Kilian 1996; Ramírez de Arellano et al. 2012). Miocene ridge collision has been proposed as a mechanism to explain the chemical variability of back-arc plateau lavas (e.g. Gorring and Kay 2001; Ramos et al. 2004; Espinoza et al. 2010). However, Ramírez de Arellano et al. (2012) showed that the presence of intrusive bodies inboard of the batholith cannot be related to ridge collision, which migrated northwards through time, as the ages of these intrusions do not change systematically from south to north. Here we investigate plutonic rocks and dikes of the Torres del Paine area, which is at the eastern edge of the Patagonian batholith (Figs. 1, 2). Magmatic rocks in this area are thus well placed to capture any eastwards migration of subduction influence. The temporal and geochemical evolution of plutonic rocks and a series of dikes from this area provide new constraints on the igneous evolution above subduction zones in response to changing geodynamic settings.

### Geology of the Torres del Paine area

The Torres del Paine area is characterized by gently folded Cretaceous sediments into which the transitional alkaline Torres del Paine intrusive complex (TPIC) was emplaced in discrete pulses from 12.6 to 12.45 Ma (Michel et al. 2008; Leuthold et al. 2012, 2013). In addition, a variety of gabbroic to granodioritic intrusive rocks dated from 29.4 ± 0.8 Ma (Altenberger et al. 2003) to 16.8 ± 0.3 Ma (Fosdick et al. 2011) predate the emplacement of the Torres del Paine intrusive complex, and basaltic dikes crosscut the entire area. The TPIC is a bimodal intrusion, which has been investigated in detail. Geochronological studies indicate that granitic magmas were emplaced over a short time interval of ~ 90 ky during the Miocene (Michel et al. 2008) and the entire Torres del Paine complex was built over 162 ± 11 ky (Leuthold et al. 2012). The geochemistry and emplacement mechanisms of the Torres del Paine intrusion have been studied by Michael (1984, 1991) and Leuthold et al. (2013, 2014). Michael favoured a model of mafic intrusions into a crystallizing granitic magma chamber. Later studies refined this model, and a piecemeal emplacement of a series of sills is advocated, based on detailed field observations and U–Pb dating on zircons. While one batch of emplacing melt is constructed of multiple pulses that show supersolidus features such as quenched mafic enclaves and/or small-scale diapirs (Leuthold et al. 2013), at least three resolvable granitic units and four mafic units can be distinguished (Michel et al. 2008; Leuthold et al. 2012). In contrast, the dike swarms and numerous smaller stocks (see Fig. 2 for a distribution of different intrusions and dikes) in the area immediately surrounding the Torres del Paine intrusive complex have not been systematically studied.

### Sample description

We have distinguished four different magmatic suites by combining field observations with petrography and geochemistry. Smaller calc-alkaline plutons (16.5–15.7 Ma) in the western part of the study area are aligned roughly north–south, while the Torres del Paine laccolith (~ 12.5 Ma) is oriented east–west (Fig. 2). Two small gabbroic sills and stocks (~ 30 Ma) have been mapped east of the Torres del Paine laccolith. The entire area is cut by basaltic to andesitic dikes that can only be distinguished from each other using geochemistry. Two dike generations are alkaline and two suites are of calk-alkaline origin.

#### Alkaline microgabbros and alkali basaltic dikes

**Fig. 3:**
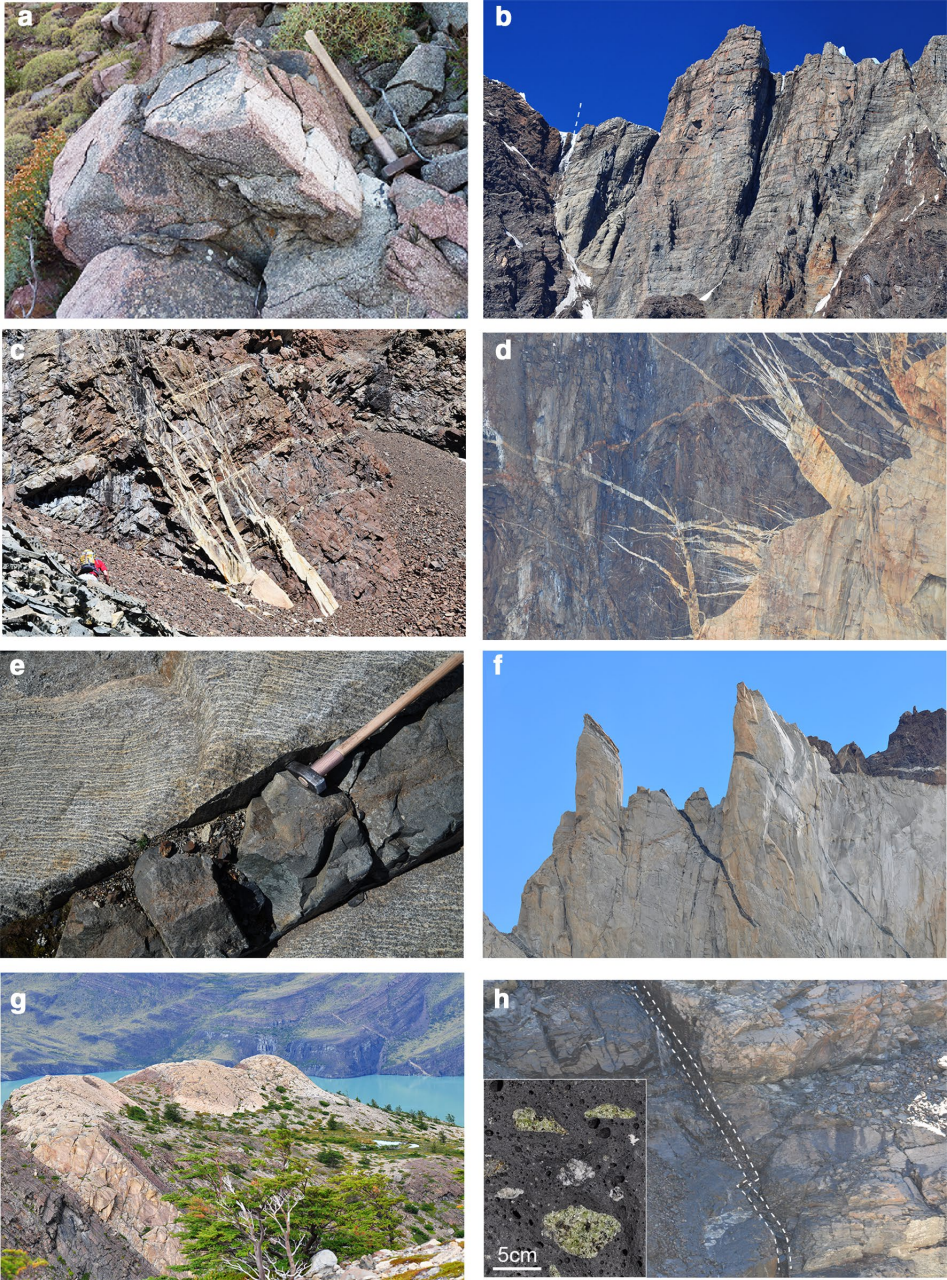
Field aspects of igneous rocks and dikes from the Torres del Paine area. **a** Monzogabbro with a doleritic texture. **b** Gabbro/Diorite stock southwest of Paine Grande with subvertical contacts to Cretaceous country rocks. **c, d** Granitic dikes (horsetail dikes) intruding Cretaceous country rocks. **e** Olivine basalt cutting layered gabbros of the Paine root zone. **f** Basaltic dike (center) and bimodal dike lower left crosscutting Paine Granite (Val Bader). **g** Bimodal dike intruding Cretaceous flysch (Lago Nordenskjöld, East of Los Cuernos). **h** Alkali basalt cutting contact metamorphic cretaceous country rocks on East ridge of Mte Almirante, inset in lower left illustrates mantle and crustal xenoliths in vesicular alkali basalt

Alkali gabbros occur as small bodies intruding the Cretaceous sediments in the east of the study area (Fig. 2) and classify as monzogabbros. They are characterized by an ophitic texture (Fig. 3a), idiomorphic plagioclase laths, and minor subhedral clinopyroxene, biotite, K-feldspar and quartz. Accessory phases are apatite, zircon and Fe–Ti oxides. Porphyritic textures are common in the basaltic dikes, yet most of the alkaline dikes are strongly altered and show partial replacement of the matrix by calcite. With the exception of a few clinopyroxenes, phenocrysts are generally not preserved.

#### Calc-alkaline plutons and dikes

Calc-alkaline plutons represent 100 m to km-scale bodies aligned along large anticlines in the western part of the studied area, ranging from the southern end of the Oldivado stock in the north, to the Passo Gardner area to the Paine Grande and Skottsberg intrusions in the south (Fig. 2). Rock types vary from gabbro/diorite (Fig. 3b) to tonalite and granodiorites. They consist of variable proportions of clinopyroxene, hornblende, normally zoned plagioclase, biotite, Fe–Ti oxides and quartz, and accessory zircon and apatite. Calc-alkaline dikes are mostly found in the western and southern part of the studied area and range from basaltic andesite to andesite. They are characterized by a porphyritic texture with plagioclase and/or amphibole phenocrysts in a fine-grained, and often strongly altered microcrystalline matrix. Amphibole phenocrysts in andesitic dikes indicate crystallization from variably hydrous magmas.

#### Torres del Paine and its dikes

The Torres del Paine complex intruded surrounding upper Cretaceous sediments and has produced a series of dikes that can be followed from the intrusions into the country rocks. Three different types of dikes can be distinguished. (1) The most spectacular ones are related to the emplacement of the various granitic sheets, and numerous horse-tail like pegmatitic and aplitic dikes and dikelets crosscut the country rocks (Fig. 3c, d). These dikes are tens to about 100 m long and their width varies from several tens of metres to mm–cm along the tips of the dikes, before ending in a series of fractures. These dikes consist of biotite, feldspars and clusters of anhedral to euhedral quartz and accessory allanite, zircon and rare tourmaline. The dikes in the host rocks developed into a miarolitic, high-silica granite with granophyric textures. (2) The Torres del Paine intrusive complex is cut by a series of m-scale basaltic to andesitic subvertical dikes with chilled margins (Fig. 3e). Rare olivine, clinopyroxene and plagioclase phenocrysts are visible in the chilled margins, but more commonly are concentrated in the centre of the dikes (Fig. 3e). These dikes are mainly found in the root zone of the Torres del Paine pluton (e.g. Leuthold et al. 2013), but some of them crosscut the Paine granites (Fig. 3f). (3) Bimodal dikes can be several metres wide and can be followed for kilometres. They are confined to the intrusion itself (Fig. 3f) and to the country rocks (Fig. 3g). They are characterized by a symmetric or sometimes asymmetric distribution of basaltic to andesitic borders and a granitic to granophyric core. The contact between the mafic and felsic parts can be either sharp or wavy. Some of the bimodal dikes are seen to cut the TPIC granites in the field (Fig. 3f). They dip towards the Paine intrusion and were described as cone sheets by Michael (1984). Two of these dikes have been sampled for U–Pb dating. Several metre thick and several kilometre long dikes crosscut the country rocks. They consist of rare biotite, plagioclase, subhedral to euhedral quartz and a eutectic intergrowth of quartz and alkali feldspar.

#### Mantle xenolith-bearing alkali basalts

These dikes are metre-scale wide and are exclusively found around Monte Almirante in the eastern part of the study area (Fig. 2). They are subvertical and crosscut the Torres del Paine pluton and its dikes and range from massive to vesicular alkali basalts. They contain olivine and clinopyroxene phenocrysts, embedded in a microcrystalline matrix of plagioclase, Ti–augite, apatite, Fe–Ti oxides and interstitial phonolitic glass. Locally they contain cm-scale mantle, crustal and sedimentary xenoliths (Fig. 3h). The field relations and the freshness of these rocks clearly distinguish them from all other alkaline dikes in the area and indicate a recent age of emplacement, similar to or younger than the 1–2-Ma-old alkaline magmas from the mantle xenolith-bearing dikes from the Cerro del Fraile (Fleck et al. 1972).

## Methodology

### Bulk rock analysis

We present 154 new major and trace element data for plutons and dikes from the Torres del Paine area. Bulk rock samples were crushed and then powdered in an agate mill, which was cleaned with quartz sand in between samples. The powders were dried overnight at 100 °C. Loss on ignition was determined by heating the samples to 1050 °C for 2 h. 1.2 g of dried powder was then mixed with 6 g of lithium tetraborate and fused at 1300 °C for 3.5 min in platinum crucibles prior to being quenched to form homogeneous glass beads. Major elements were measured on lithium tetraborate glasses and acquired by X-ray fluorescence using a Philips PW 2400 spectrometer at the University of Lausanne (UNIL). The standards SY-2, NIMN, NIMG, BHVO and BE-N (Govindaraju 1994) were used as quality control. Absolute uncertainties in XRF analyses are in the range of 0.5 wt% (2*σ*) for major elements such as SiO_2_ to < 0.01 wt % for minor elements.

Whole rock trace elements were measured using an ELEMENT XR single collector, sector-field, inductively coupled plasma mass spectrometer (SF-ICP-MS) interfaced to a NewWave UP-193 ArF excimer ablation system at UNIL. Trace elements concentrations were measured on the flat side of XRF glass beads. Analytical conditions were 10–15 Hz repetition rate, an energy of ~ 160 mJ, which is equivalent to 12 J/cm^2^, and a 100 µm spot diameter. Helium was used as a carrier gas. At least three repeat measurements were performed on each sample, with average standard deviations of 5.2% for Sc, 7.0% for Ce and 15.5% for U, with Cs reaching the highest value of 39.7% (2*σ*). Background and laser ablation signal acquisition times were ~ 100 and 50 s, respectively. Dwell times for the different isotopes range from 10 to 20 ms employing a peak-hopping mode. The analytical setup was tuned for optimal conditions for the entire mass range and oxide production rates were assured to be below 0.2% determined by monitoring the Th/ThO intensity ratio < 0.002. Two analyses of NIST SRM 612 at the beginning and end of each analytical series were used to bracket up to 16 analyses of unknowns and to correct for drift of the instrument. The blank concentration was determined before each analysis and its spectrum was subtracted from the spectrum of the analyte. Absolute trace element concentrations were determined using CaO previously measured by XRF as an internal standard. For trace element quantification the preferred values from the external standard NIST SRM612 were used (Pearce et al. 1997). Data were processed using LAMTRACE (Jackson 2008).

### U–Pb geochronology

Samples were crushed and zircons were extracted using density (panning) and magnetic techniques. Zircons were handpicked and mounted in 2.5-cm epoxy discs, polished to expose their mid-sections, and imaged in cathodoluminescence (CL). CL images were taken on a CamScan MV2300 scanning electron microscope at UNIL, with 10 kV accelerating voltage, probe current of 0.7 nA and a working distance of 45 mm. U–Pb ages were measured on the same LA-ICPMS as for trace elements but with a spot size of 35 or 50 µm, a repetition rate of 20 Hz, and energy density on the sample of 2.3 J/cm^2^.

A single megacryst of GJ-1 zircon was used as the primary standard for U–Pb geochronology. Our crystal of GJ-1 has a ^206^Pb/^238^U age of 600.5 ± 0.4 Ma as determined by TIMS analysis at the University of Geneva (Schaltegger, personal communication). A natural zircon was analysed as a secondary standard to monitor data accuracy, either 91,500 (1065 Ma, Wiedenbeck 1995) or Plešovice (337.13 ± 0.37 Ma; Sláma et al. 2008).

U–Pb data were treated using the LAMTRACE program (Jackson 2008) and the ratio-of-the-mean intensity method (Ulianov et al. 2012). U–Pb data were not corrected for common Pb. The large isobaric interference from the Hg that is ubiquitously present in Ar used to transport the analyte in ICPMS makes accurate measurement of ^204^Pb difficult, and a ^204^Pb correction for Pb_c_ impractical (Horn et al. 2000; Andersen 2002; Gerdes and Zeh 2006). Most zircon analyses that are unaffected by inclusions or metamictisation have negligible common Pb (e.g. Horn et al. 2000) and a typical approach in U–Pb geochronology by ICPMS is not only to apply no correction for common Pb, but to exclude any discordant analyses (Horn et al. 2000; Gerdes and Zeh 2006). We adopt this approach and include only concordant analyses in the calculation of sample ages, noting that taking the lower intercept of a linear regression through all analyses on a Tera–Wasserburg concordia always gave indistinguishable ages (see online appendix Section S2).

IsoPlot Ex 3.5 (Ludwig 2003) was used to plot data on Tera–Wasserburg concordia diagrams (Tera and Wasserburg 1972) and to calculate weighted mean ages. The final uncertainty on sample ages was calculated by adding in quadrature the 95% confidence interval on the weighted mean calculated for the sample by Isoplot, and the reproducibility (2 S.D.) of GJ-1 in the same session (Table S2). Reproducibility of GJ-1 was 0.7–1.9% (2 SD) in the four sessions, without exclusion of any primary standard analyses (Tables S2, S4). In each session, the secondary standard 91500 or Plešovice gave a weighted mean ^206^Pb/^238^U age that was statistically indistinguishable from its nominal value once error propagation was performed (Table S3). Over four analytical sessions, a total of six analyses (out of 93) of the secondary standards were excluded, five for being discordant and one whose age was a clear outlier.

## Results

### Bulk rock chemistry

**Fig. 4:**
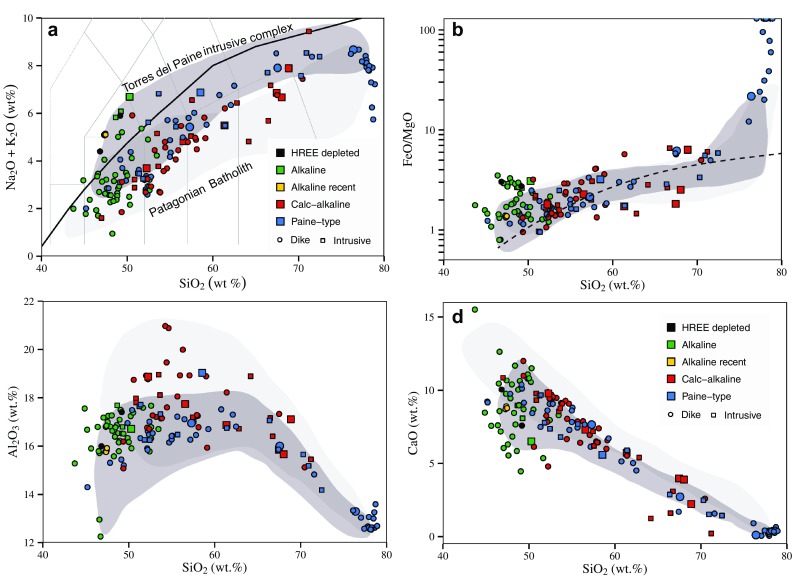
Major element variation diagrams illustrating the different fractionation trends of the Torres del Paine rocks. **a** TAS diagram after LeBas et al. (1986). Thick black curve indicates the alkaline–subalkaline boundary after Irvine and Baragar (1971). **b** FeO_tot_/MgO vs SiO_2_, with field boundary tholeiitc vs calc-alkaline after Miyashiro (1974). **c** SiO_2_ vs Al_2_O_3_, note that the calc-alkaline rocks plot on average at higher Al_2_O_3_ for a given SiO_2_ indicating that fractionation is at higher pressures (Müntener and Ulmer 2018). **d** SiO_2_ vs CaO, all samples plot along a straight line from andesitic to rhyolitic compositions. Larger symbol size indicates the selected samples dated by U–Pb on zircons. Torres del Paine field compiled from Michael (1984) and Leuthold et al. (2013). Patagonian batholith from Hervé et al. (2007), ranging from late Jurassic to the Neogene

Based on whole rock geochemistry, dikes and plutons are divided into three main groups (Fig. 4): “alkaline”, with chemistry straddling the alkaline–subalkaline boundary in the total alkali–silica (TAS) diagram, which includes also the alkaline recent-age dykes; “calc-alkaline”, plotting in the subalkaline field on the TAS diagram and following a typical calc-alkaline differentiation trend on a plot of FeO/MgO vs SiO_2_; and “Paine-type”, which show a high K calc-alkaline whole rock chemistry intermediate between the calc-alkaline and alkaline groups, similar to the chemistry of the TPIC itself (Leuthold et al. 2013). Finally, two dikes with strong heavy REE depletion are distinguished from all other dikes.

Whole rock compositions of alkaline rocks for the investigated area broadly fall into two different groups. The first group includes monzogabbros and basaltic dikes. This group straddles the alkaline–tholeiitic boundary in the TAS diagram (Fig. 4a). The relatively low total alkali content of most of the alkaline rocks is related to significant alteration as reflected by elevated loss on ignition (LOI) for many of these samples (Electronic Appendix, Table S1). The fresh samples from the youngest suite of alkaline dikes represent alkali basalts. All other dikes generally follow a calc-alkaline differentiation trend (Fig. 4b). For intermediate compositions, FeO/MgO remains in a narrow range (2–6) over a wide range of SiO_2_ (55–71 wt%), typical for calc-alkaline series, while CaO monotonously decreases from values around 10 wt% to less than 1% with increasing SiO_2_ (Fig. 4d). In terms of major elements, the SiO_2_ vs Al_2_O_3_ diagram illustrates best the differences between the calc-alkaline series and the high K transitional Paine magmatic rocks (Fig. 4c), while the alkaline rocks are most distinct in silica vs incompatible trace element diagrams.

**Fig. 5:**
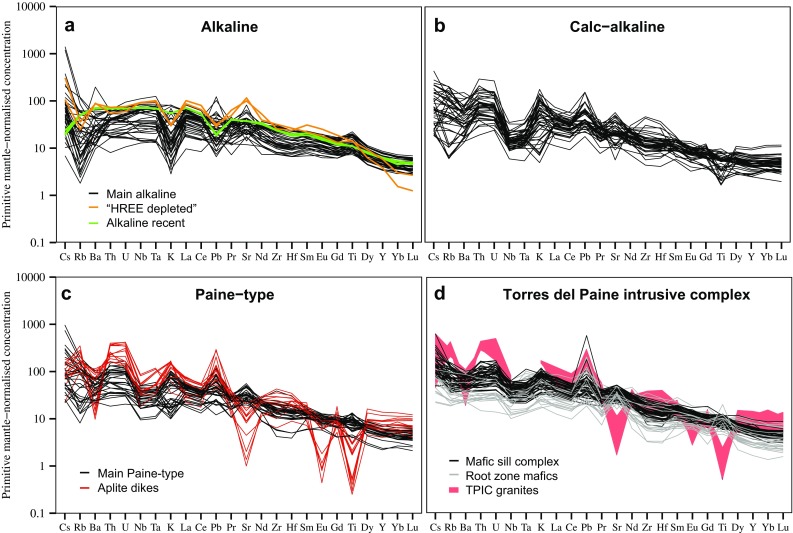
Primitive mantle normalized trace element diagrams for plutonic rocks from the Torres del Paine area: **a** alkaline gabbros and dikes. **b** Calc-alkaline plutons and dikes. **c** Dikes and small intrusions related to the Torres del Paine magmatic system. **d** Compiled data for the Torres del Paine laccolith, data from Leuthold et al. (2013), Michael (1991) and our own unpublished data. Normalization values from McDonough and Sun (1995)

**Fig. 6:**
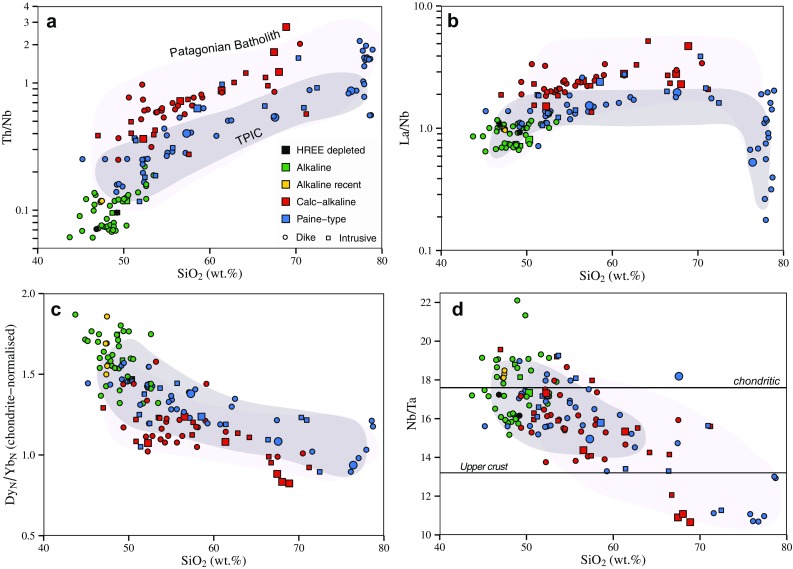
**a** Variation in SiO_2_ vs Th/Nb in bulk rock data from the Torres del Paine area (data from this study and Leuthold et al. 2013; Hervé et al. 2007). Both the calc-alkaline and the Torres del Paine series increase with increasing SiO_2_ but the values at 50 wt% SiO_2_ are clearly different. **b** Variation of La/Nb vs SiO_2_, calk-alkaline series remain mostly above 2, while alkaline rocks vary around 1. Torres del Paine rocks are intermediate between the two. **c** Variation of SiO_2_ vs Dy/Yb, a proxy for amphibole and/or garnet fractionation in comagmatic rock series (Davidson et al. 2007). Both the calk-alkaline and Paine series decrease with increasing SiO_2_, indicating that amphibole fractionation exerts an important control on both series. Note that the two HREE-depleted samples plot above the range shown here. **d** SiO_2_ vs Nb/Ta ratios for the different magmatic rocks of the Torres del Paine area. Note that the alkaline rocks show a large variability of Nb/Ta for a SiO_2_ range between 50 and 55. Both the calc-alkaline and the Paine-type rocks display a weak decrease in Nb/Ta with increasing SiO_2_, consistent with the range of the Patagonian batholith (Hervé et al. 2007). Chondrite value from Sun and McDonough (1989), upper crustal value from Rudnick and Gao (2003)

With the exception of K_2_O and Rb, primitive mantle normalized trace element patterns for the alkaline rocks resemble OIB (Fig. 5a), and are characterized by positive Pb and Ti anomalies. Two dikes of this group display high Sr/Y and La/Yb ratios, they have less than 50 wt% SiO_2_, low Mg# (~ 0.4) and no HFS element negative anomaly. The calc-alkaline plutons and dikes and the high-K Paine magmatic series have chemistry typical of subduction-related rocks, as shown by low Nb, Ta contents and enriched highly incompatible trace elements pattern (Rb, Ba, Th, U) (Fig. 5b, c). Some of the intrusions from the Skottsberg and Oldivado areas have concave downward REE patterns, indicating hornblende fractionation. A few samples (mainly aplitic to rhyolitic dykes) of the Paine series have negative Ti, Eu, Sr and Ba anomalies indicating plagioclase + Fe–Ti oxide fractionation (Fig. 5c). Although the Paine series and the calk-alkaline series are quite similar in their overall trace element patterns, in detail, some trace element ratios allow for a clear distinction of the two series (Fig. 6). The most prominent geochemical difference between the two series is the higher Th/Nb ratio for a given SiO_2_ for the calc-alkaline series (Fig. 6a). High La/Nb and high Th/Nb ratios are characteristic for rocks with negative Nb anomalies in arc rocks, independent of any alkali or LILE element enrichment. A similar difference between the calc-alkaline series and the Paine dikes can be seen on diagrams of Dy/Yb vs SiO_2_ (Fig. 6b) and La/Nb vs SiO_2_ between about 50 and 70 wt% SiO_2_ (Fig. 6c). The calc-alkaline series generally have a lower [Dy/Yb]_*N*_ ratio between 50 and 70 wt% SiO_2_. The La/Nb ratio at about 50 wt% SiO_2_ is generally > 1 for monzogabbros and alkali basalt, while the calc-alkaline rocks are generally > 2. The high-K Paine series is intermediate between the alkali dikes and monzogabbros, and the calc-alkaline series, except for low values of silica-rich rocks (SiO_2_ > 75 wt%).

### U–Pb geochronology

**Fig. 7:**
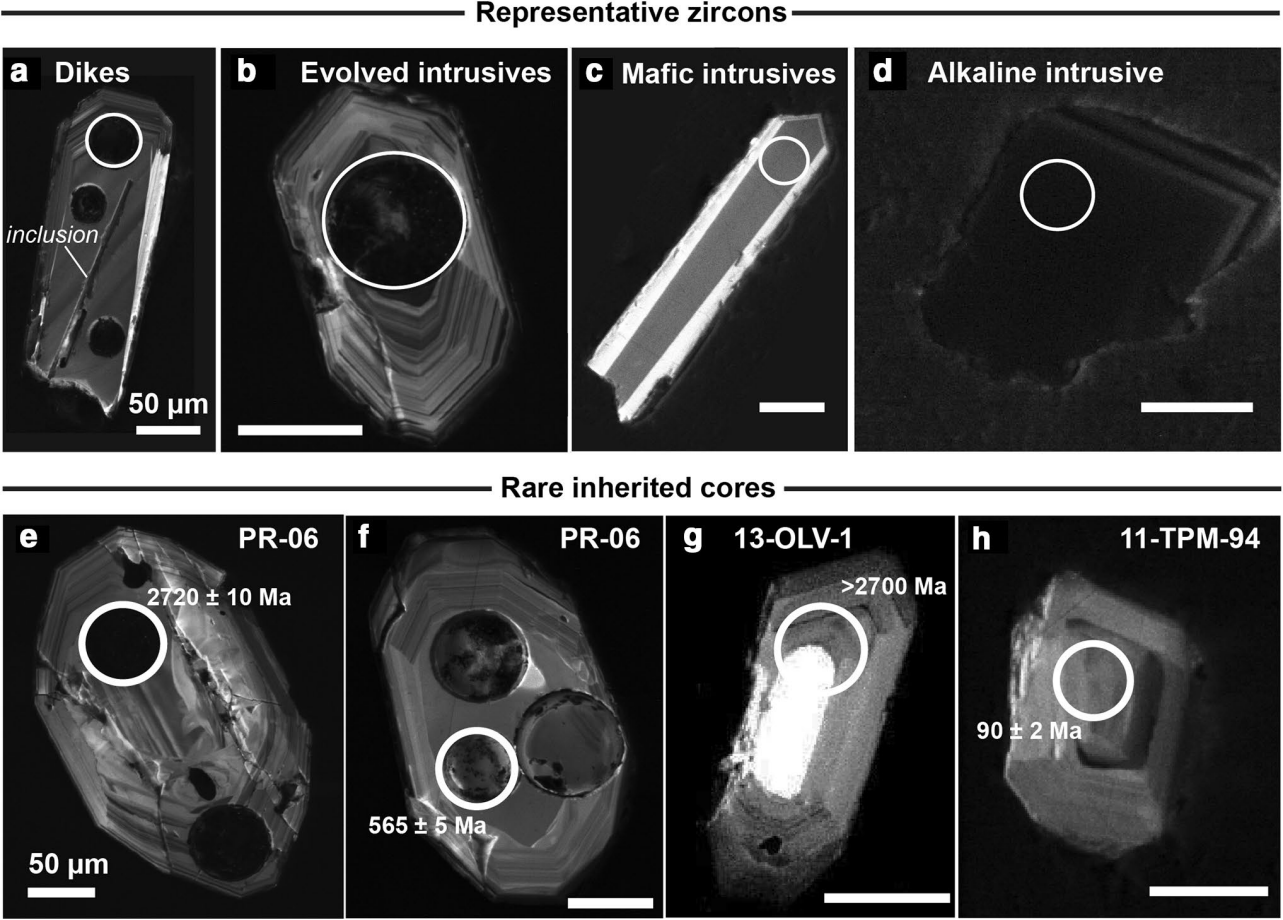
Representative cathodoluminescence (CL) images of zircons from the dated rocks (**a**–**d**) and rare inherited core domains (**e**–**h**). U–Pb laser spots are indicated. **a** 11-TPM-32A, **b** PR-06, **c** 11-TPM-61A, **d** 11-TPM-73A

Zircons from twelve samples were U–Pb dated. Zircons display oscillatory cathodoluminescence (CL) zoning, homogeneous CL emission, or faint panelled zoning (Fig. 7a–d). In four samples rare discordant or partially resorbed cores are observed in CL (Fig. 7e–h), but in the other eight samples there is no evidence in CL for more than one generation of zircon growth. Zircon characteristics in transmitted light and CL are described in detail in Section S1.

Many zircons contain abundant inclusions (Section S1) that were difficult to avoid during laser ablation. Thirty-five analyses (from a total of 289) with irregular laser ablation time-resolved elemental concentration spectra or for which post-analysis inspection revealed sampling of inclusions were excluded a priori as contaminated or mixed between two age zones. These analyses are not shown in data tables or plots.

**Fig. 8:**
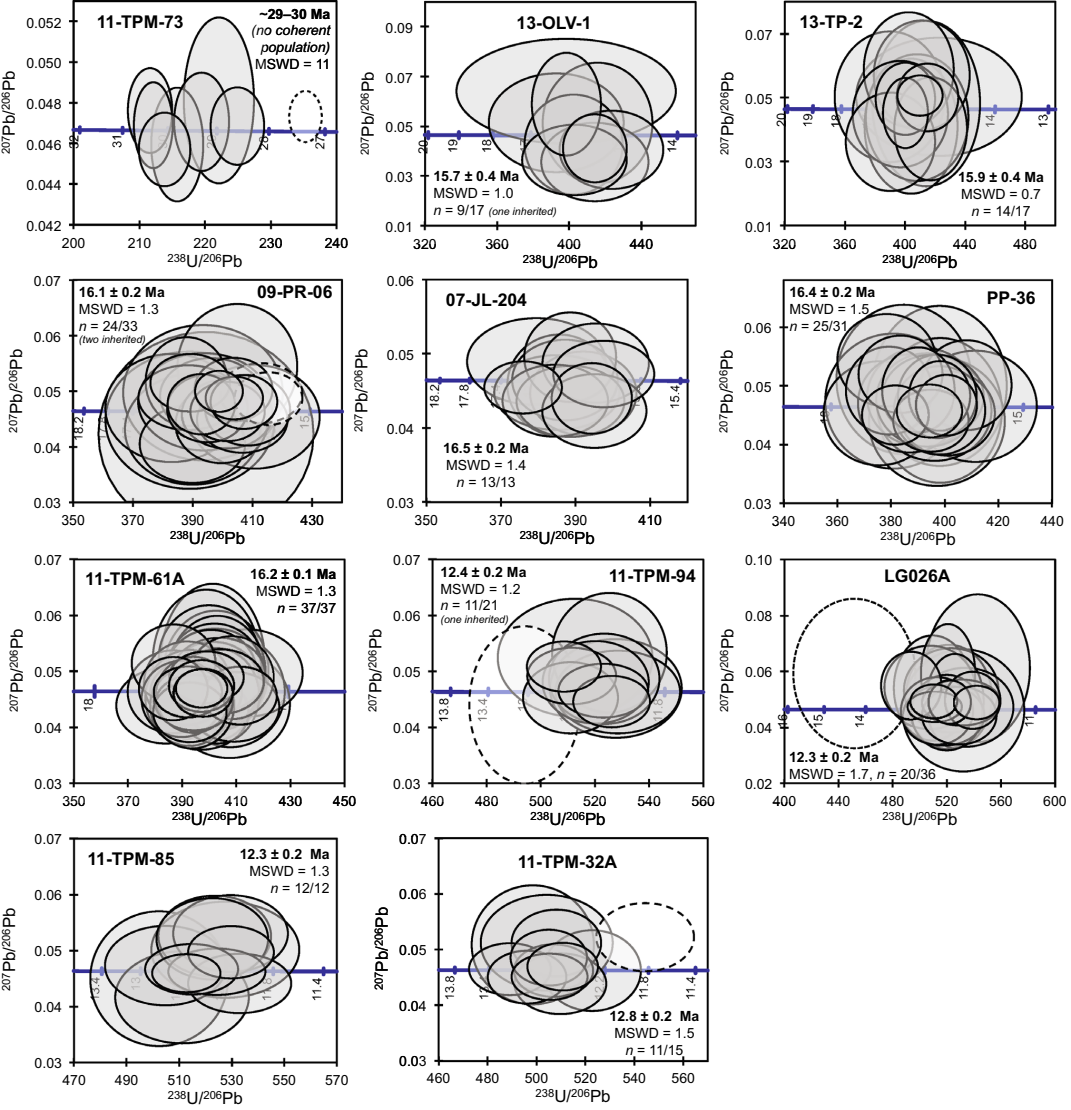
Tera–Wasserburg concordia diagrams showing U–Pb data used to calculate sample ages for eight intrusive and three dike samples from the Torres del Paine region. Data are plotted uncorrected for common Pb. Analyses excluded as age outliers are dashed. The weighted mean ^206^Pb/^238^U age and its MSWD are given as text. *n* indicates the number of analyses used to calculate the age, as a fraction of total analyses; all analyses including those excluded due to high common Pb or inclusions are plotted on Tera–Wasserburg diagrams in Fig. S4. Bar plots of ^206^Pb/^238^U dates with the sample weighted average are given in Fig. S5

**Fig. 9:**
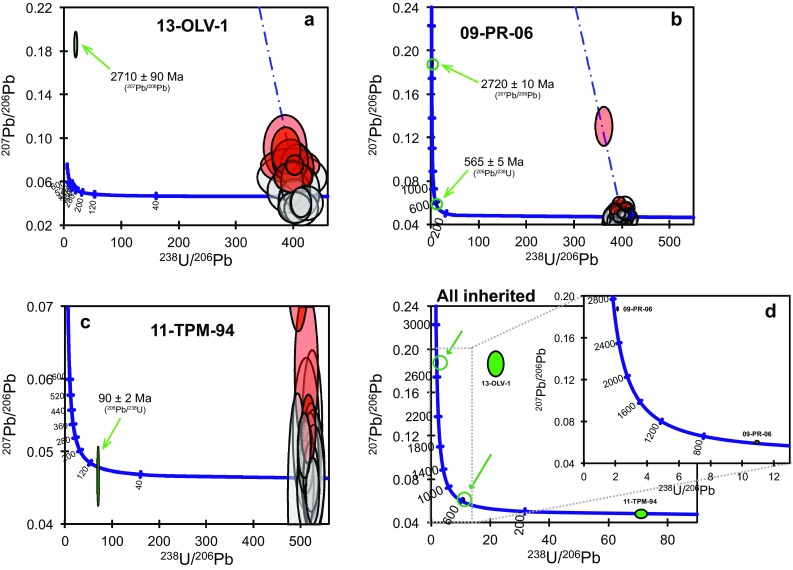
Tera–Wasserburg concordia diagrams, uncorrected for common Pb for (**a**–**c**) inherited zircons from three samples, with igneous concordant (grey) and discordant (red) analyses for comparison. Blue dashed lines show regression through concordant and discordant igneous analyses towards common Pb; inherited ages are clearly unrelated to these trends. Preferred ages given as text for each inherited analysis. **d** Inherited ages only, compiled from three samples. Green circles and arrows highlight two analyses visible in the inset

For most samples analysed, the remaining U–Pb dates form arrays ranging from concordant to increasingly discordant along a single trend towards increasing proportions of common lead (Pb_c_; Fig. S4). In most cases, high-Pb_c_ analyses were associated with domains that were cracked or damaged, or zircons that were rich in inclusions. These discordant analyses are interpreted as contaminated to varying degrees by ‘foreign’ common Pb introduced by sampling of inclusions that could not be observed during post-analysis inspection (e.g. because the entire inclusion was ablated) or introduced into cracks during sample preparation (Online Appendix section S2). Two analyses gave extremely imprecise dates (> 30% 1 sigma on the ^207^Pb/^206^Pb), which are interpreted to result from contamination by inclusions. All discordant and imprecise analyses were excluded from the calculation of sample ages and are not presented in Figs. 7, 8 and 9, but are given in Table S6 and plotted in Fig. S4. A detailed description of the U–Pb data for each sample, including discordant data, is given in Section S1. Apart from rare inherited cores, U–Pb data defined a single population in each sample, with MSWDs close to 1. Ages are interpreted to date the crystallization of each magmatic body.

#### Intrusive bodies

Zircons from the monzogabbro 11-TPM-73A have CL and transmitted light characteristics suggesting some are strongly radiation damaged. In transmitted light many are extremely dark and appear to be highly damaged, often associated with unusual ‘patchy’ CL emission. Most zircons are very dark in CL. Of 13 analyses, eight were concordant. Of these, one analysis of a zircon with high Th gave a distinctly younger date attributed to Pb loss due to radiation damage. The remaining seven concordant analyses still have very scattered ^206^Pb/^238^U dates, with an MSWD of 11 (Fig. 8). Many zircons from this sample had extremely high counts on Th and U isotopes, indicating dramatically higher Th and U concentrations relative to other samples. These zircons are, therefore, likely to have suffered radiation damage and associated Pb loss. This may have contributed to the scatter in dates observed for this sample. The age of this sample is constrained only approximately to ~ 29–30 Ma, defined by seven analyses that are concordant but show excess scatter (MSWD = 11). This age is nonetheless clearly distinguished from the age of all other samples from this study, and is in line with a K–Ar age of a similar gabbro (29.4 ± 0.8 Ma, Altenberger et al. 2003).

Of seventeen zircons from the calc-alkaline Oldivado intrusion (13-OLV-1), nine analyses were concordant and gave a weighted mean ^206^Pb/^238^U age of 15.7 ± 0.4 Ma with an MSWD of 1.0 (Fig. 8). The quoted uncertainty for this and all other weighted means is the external uncertainty, i.e. with the 2 SD of the standard already propagated (see Table 1 for internal uncertainties). One analysis of 13-OLV-1 was partly on a CL-bright core domain, but partly sampled the surrounding oscillatory-zoned rim (Fig. 7a), and gave a strongly discordant date that is interpreted as a mixed core–rim age. This analysis does not have significantly higher ^208^Pb than other analyses from the same sample, indicating that there is little or no common Pb contribution. Its ^207^Pb/^206^Pb date of 2710 ± 90 Ma is, therefore, taken as a minimum age for the inherited core. The strong discordance of this analysis, with a ^206^Pb/^238^U date of 290 ± 30 Ma, is at least in part due to mixing with the much younger rim domain, although partial Pb loss may also have affected it.

**Table 1:** Sample U–Pb ages

Sample	Unit	Lithology	Session	2 SD of GJ-1 (%)	Weighted mean ^206^Pb/^238^U age (Ma)	± 95% conf. internal	External error (Ma)	MSWD	*n*	Inherited cores
Intrusive bodies										
11-TPM-73A	Amarga	Gabbro	3	1.1	~ 29–30 Ma	–	–	11	7/13	
13-OLV-1	Olvidado	Monzogranite	4	1.9	15.7	0.3	0.4	1	9/17	> 2710 ± 90 Ma
13-TP-2	Gardner	Granodiorite	4	1.9	15.9	0.2	0.4	0.7	14/17	
09-PR-06	Lago Grey/Paine Grande?	Granite	1	0.7	16.1	0.1	0.2	1.3	24/31	2720 ± 10, 565 ± 5 Ma
07-JL-204	Upper Skottsberg	Granodiorite	2	0.9	16.5	0.2	0.2	1.4	13/13	
PP-36	Lower Skottsberg (Tocardo?)	Granitic pegmatite	1	0.7	16.4	0.2	0.2	1.5	25/31	
11-TPM-61A	Almirante	Diorite	3	1.1	16.2	0.1	0.2	1.3	37/37	
11-TPM-94	Bader	Monzonite	3	1.1	12.4	0.1	0.2	1.2	11/21	90 ± 2 Ma
Dikes										
LG-026A	Felsic dikes	4	1.9	12.3	0.1	0.2	1.7	20/36		
LG-003B*	Bimodal dikes	Felsic (dated)/andesitic	4	1.9	12.6	0.3	0.4	0.5	9/9	
11-TPM-85	Felsic dikes	Granitic	3	1.1	12.3	0.1	0.2	1.3	12/12	
11-TPM-32A	Bimodal dikes	3	1.1	12.8	0.2	0.2	1.4	11/15		

*n* indicates the number of analyses used in calculation of the sample age as a fraction of the total number of analyses. External error is that including the propagated reproducibility of GJ-1, and is the preferred age uncertainty

Of 17 analyses of zircons from sample 13-TP-2 (small intrusion at Paso Gardner, Fig. 2), 14 analyses were concordant and gave a weighted mean ^206^Pb/^238^U age of 15.9 ± 0.4 Ma with an MSWD of 0.7 (Fig. 8). Two analyses of zircons from sample 09-PR-06 (Granite SW of Paine Grande, Fig. 2) were on partially resorbed or replaced central domains (Fig. 7b, c) that are interpreted as inherited cores. One gave a concordant date with a ^206^Pb/^238^U age of 565 ± 5 Ma (Fig. 9b). The other was subconcordant with a ^207^Pb/^206^Pb date of 2720 ± 10 Ma and a ^206^Pb/^238^U date of 2520 ± 20 Ma (Fig. 9b). The small difference in age is interpreted to result from a low degree of Pb loss, and the ^207^Pb/^206^Pb age of 2720 ± 10 Ma best records the age of this inherited domain. Of the remaining 31 analyses, 26 were concordant and two analyses of very inclusion-rich zircons gave distinctly younger ages and were excluded (Fig. 8). The remaining 24 analyses gave a weighted mean ^206^Pb/^238^U age of 16.1 ± 0.2 Ma, with an MSWD of 1.3. All 13 analyses of zircons from 07-JL-204 (SE of Punta Bariloche, Fig. 1) were concordant (Fig. 8), and gave a weighted mean ^206^Pb/^238^U age of 16.5 ± 0.2 Ma with an MSWD of 1.4. Of 31 analyses of zircons from sample PP-36 (granite SE of Punta Bariloche, Fig. 1), 25 were concordant (Fig. 7) and gave a weighted mean ^206^Pb/^238^U age of 16.4 ± 0.2 Ma with an MSWD of 1.5. Thirty-seven analyses of zircons from 11-TPM-61A were concordant dates and gave a weighted mean age of 16.2 ± 0.2 Ma with an MSWD of 1.3 (Fig. 8). Of 21 analyses of zircons from 11-TPM-94 (a small monzodiorite intrusion at the entrance of Val Bader), one analysis of a central domain (Fig. 7d) was concordant but significantly older with a ^206^Pb/^238^U date of 90 ± 2 Ma (Fig. 9c), and is interpreted as an inherited core. Of the remaining analyses, 12 were concordant. One analysis of a zircon with abundant inclusions was a distinct outlier in age (Fig. 8), and was excluded. The remaining 11 analyses gave a weighted mean age of 12.4 ± 0.2 Ma, with an MSWD of 1.2.

#### Dikes

Of 36 analyses of zircons from LG-026A, 21 were concordant. One which was a distinct outlier in ^206^Pb/^238^U age (Fig. 7) was excluded as probably contaminated by a sub-surface inclusion. The remaining 20 analyses give a weighted mean ^206^Pb/^238^U age of 12.3 ± 0.2 Ma with an MSWD of 1.7. Twelve analyses of zircons from 11-TPM-85 were all concordant and gave a weighted mean ^206^Pb/^238^U age of 12.3 ± 0.2 Ma with an MSWD of 1.3 (Fig. 7). Of 15 analyses of zircons from 11-TPM-32A, three on inclusion-rich zircons were excluded as discordant or very imprecise (Fig S3), and one analysis distinctly younger in ^206^Pb/^238^U age, also on an inclusion-rich zircon, was excluded (Fig. 8). The remaining 11 analyses give a weighted mean age of 12.8 ± 0.2 Ma, with an MSWD of 1.5.

## Discussion

There are variations in the geochemical signatures of the studied rock series in the Torres del Paine area as a function of the age of emplacement of the rocks. An important issue is what causes these variations and how they may be linked to the geodynamic evolution of the southern Andean subduction system. This question can be addressed by evaluating two parameters: one is based on the slab component that mainly affects the incompatible trace element composition and ratios of the studied rocks, and one is based on the effects of fractionation from primitive basaltic magmas to evolved granitoids. (1) The slab component is primarily derived from dehydrating and/or melting of the subducting slab. A number of trace element ratios have been invoked to be excellent tracers of the slab component, such as Ba/Nb, Th/Nb, Th/La or LILE to LREE ratios (e.g. Elliott et al. 1997; Plank 2005; McCulloch and Gamble 1991). Variations in the amount of subducted sediment contribution to arc magmas will lead to large differences in trace element ratios of elements with similar compatibility during mantle melting, such as Th/Nb, La/Nb or Ba/Th. These ratios are, therefore, relatively insensitive to the degree of melting. (2) The crystal fractionation process can be identified in coherent rock series that show a large variation in a number of major element concentrations such as SiO_2_ vs Mg#, CaO or Al_2_O_3_. Major elements coupled with trace element ratios that are sensitive to the fractionating assemblage, such as Ba/Sr or Dy/Yb (e.g. Davidson et al. 2007) are a powerful approach to identify intracrustal fractionation processes. For the case of the Torres del Paine area we first evaluate the significance of inherited zircon cores, before discussing in detail the source variations and phase control on fractionating assemblages.

### Recycling of ancient components as documented from a few inherited zircons

An important result from our zircon data is that only three of the 12 samples contain inherited zircon cores, in spite of the large number of analyses for some samples and the targeting of apparent core domains whenever they were observed in CL. The distinct lack of inherited domains is corroborated by CL images, which show no evidence for more than one generation of zircon growth in the vast majority of grains (Fig. 7). Even in the samples where inherited cores were found, they are rare. The overall paucity of inherited zircon suggests that crustal assimilation was probably limited for these magmas and that the geochemical trends can be interpreted as a combination of slab-derived components and fractionation processes.

The presence of dated inherited domains with ages of up to ~ 2700 Ma in two samples from the calc-alkaline series (Fig. 9d) demonstrates the existence of ancient continental crust below the Torres del Paine region, in spite of the fact that such old crust is not exposed at the surface. The two samples that contain inherited zircons older than the Patagonian batholith (500–2700 Ma; 13-OLV-1 and 09-PR-06) are among those belonging to the calc-alkaline series and the most evolved rock types dated, implying a longer residence time in the crust and thus favouring crustal assimilation for these samples. The fact that ancient zircon-bearing crust is not exposed at the surface in the greater Torres del Paine region indicates that this assimilation must have occurred at depth in the crust. A large dataset from the entire Patagonian batholith shows only a few inherited zircon grains in 9 out of 64 samples that are older than the Patagonian batholith (Hervé et al. 2007) but no zircon was reported with ages > 2 Ga. These zircons, which undoubtedly indicate assimilation processes of old basement rocks at depth, are however, extremely rare, and will not be considered further in this paper.

### Origin of the different magmatic series in the Torres del Paine area over time

The plutons and dikes from the Torres del Paine area can be classified into two major groups, one is predominantly alkaline and the other calc-alkaline arc rocks. With the exception of the young alkali basaltic dikes most of the alkaline rocks have lost considerable amounts of alkalis during alteration as illustrated by negative Rb and K anomalies on the spider diagrams (Fig. 5a). This loss of alkali elements is the reason that many alkali basalts plot in the tholeiite field in the TAS diagram (Fig. 4a). Other than fluid mobile elements the trace element characteristics of the alkaline rocks resemble OIB in many respects (e.g. high Nb–Ta, positive Ti anomaly, high La/Yb ratios). The similarities of the trace element patterns between the monzogabbroic rocks and the alkaline dikes indicate a common origin and point to OIB-like magmatism at ~ 29–30 Ma consistent with a K–Ar age for alkali gabbro sills in the Cretaceous sediments (Altenberger et al. 2003). Alkali gabbros and dikes are mainly found in the eastern part of the study area, consistent with a rear-arc position at that time.

The calc-alkaline (~ 16–17 Ma) and Paine-type (~ 12.5 Ma) series of plutons and dikes from the Torres del Paine area are calc-alkaline to high-K transitional arc rocks. The trace element characteristics with Nb and Ta depletion, and LILE and Pb enrichment indicate a clear subduction zone signature for both the calk-alkaline and the high-K transitional arc rocks. Pronounced negative Ti, Ba, Sr and Eu anomalies indicate fractionation of plagioclase for the most evolved granitic rocks, mostly aplitic dikes (Fig. 5c). Trace element ratios such as Th/Nb and La/Nb, however, indicate important differences between the calc-alkaline and the transitional Paine series. Both Th and Nb have bulk partition coefficients for mantle melting < 0.01, so their ratio is almost unaffected even by low degrees of partial melting, and is, therefore, very sensitive to the addition of a mobile component from the subducted slab (Elliott et al. 1997). While most of the alkaline rocks have Th/Nb ratios less than 0.14, similar to MORB, higher Th/Nb ratios are characteristic for the calc-alkaline rocks (Fig. 6a). Both the calc-alkaline plutons and the high-K transitional plutons and dikes show good correlations of Th/Nb with increasing SiO_2_. Simple linear regression of the Paine magmatic rocks indicates that Th/Nb ratio at 50 wt% SiO_2_ is around 0.2 ± 0.02, similar to the Greater Antilles arc and the intraoceanic Mariana and Tonga arcs (Kelemen et al. 2003). The Th/Nb ratio of the calc-alkaline series is higher and range from about 0.4 to 1.1, whereas most of the alkali basalts of the Paine area show Th/Nb < 0.14 (Fig. 6a). By taking the Th/Nb ratio at 50 wt% SiO_2_ as a proxy for the slab component prior to differentiation in the two rock series (Fig. 6a), the decrease of the Th/Nb ratio from ~ 0.4 at ~ 16 Ma in the calc-alkaline series to ~ 0.2 at 12.5 Ma in the Paine-type series can be related to a decrease of the slab derived signature in the period from ~ 16.5 to 12.5 Ma.

A remarkable observation is that the ~ 29–30-Ma-old monzogabbros and dikes, and the recent alkaline basaltic dikes show higher total alkali content, and incompatible trace element ratios that are different from the Miocene calc-alkaline dikes and plutons, as illustrated by the Th/Nb and Th/La ratios (Fig. 6). In fact, ~ 30 Ma and recent alkaline rocks display trace element patterns and ratios that are typical for the rear-arc alkaline plateau lavas in Southern Patagonia, with Th/La ratios mostly below 0.15, and not exceeding 0.2 (e.g. Gorring and Kay 2001). The Torres del Paine area is thus affected by rear-arc alkaline magmatism at ~ 30 Ma and recent times that bracket a period of arc magmatism between ~ 17 and 12.5 Ma. This indicates that in one and the same area, magma characteristics changed from rear-arc alkaline to calc-alkaline between ~ 30 and 16.5 Ma. Such a change in magma characteristics during the Miocene has been related to eastward arc migration, some 200 km further north of the Torres del Paine area in the Chalten–Fitz Roy complex (e.g. Ramírez de Arellano et al. 2012). Then, ‘waning’ of the subduction signature by ~ 12.5 Ma and back to recent alkaline magmatism suggests that active subduction migrated back towards the west at present times, as indicated by active volcanism about 25 km to the west of the study area (Fig. 1). Our time-resolved trace element data show a strong increase followed by a decrease of the subduction component for magmas emplaced in the Torres del Paine area. A model in which the chemical systematics of the Paine area is primarily regulated by different extents of melting of the mantle wedge coupled to an increase followed by a decrease in the slab component over the last 30 my is the most appealing to interpret the data.

#### Modelling trace element fractionation during differentiation: the case of Nb/Ta and Dy/Yb

**Fig. 10:**
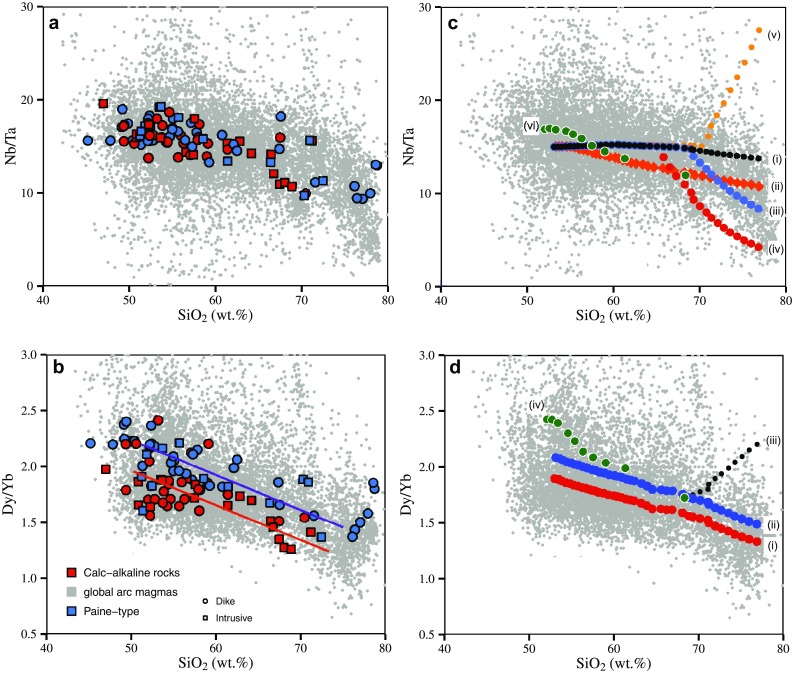
SiO_2_ vs Nb/Ta (**a**) and SiO_2_ vs Dy/Yb (**b**) for global arc magmas, our data and simple models of crystal fractionation (**c, d**). Data were filtered to exclude rocks that have SiO_2_ < 40 wt%, TiO_2_ > 4 wt%, Al_2_O_3_ > 22 wt%, Ta < 0.05 ppm, Dy/Yb > 10 and Nb/Ta > 100 and loss on ignition LOI > 5 wt%. Modelling parameters and results are listed in Table S7 and shown in steps of 3% crystallization of (model i): hornblende only; model (ii): hornblende + plagioclase in proportions as in experiments of Nandedkar et al. (2014), joined by 5% biotite at 55 wt% SiO_2_; (model iii): as model (ii), but joined by 70% plagioclase and 30% biotite at 70 wt% SiO_2_; (model iv): as model (ii) but joined by 50% plagioclase and 50% biotite at 65 wt% SiO_2_; model (v), as model (ii) but joined by 3% titanite at 70 wt% SiO_2_. Liquid lines of descent modelling of high-K arc magmas in Mongolia (model vi), with data from Bucholz et al. (2014). SiO_2_ vs Dy/Yb: modelling results are shown for steps of 3% crystallization of amphibole + plagioclase ± apatite in proportions as in experiments of Nandedkar et al. (2014). The red and blue dots are only distinguished by their initial Dy/Yb ratio of 1.9 and 2.1, respectively. In the 3rd model, 1% of zircon was added to the fractionating assemblage at 69 wt% SiO_2_, to illustrate the effect of mineral fractionation with a low Dy/Yb ratio. In model (iv) Dy/Yb was modelled using the liquid line of descent mass balance of Bucholz et al. (2014). The consistency of the Paine data and the global arc magma array indicates that amphibole + biotite are the dominant minerals in producing silica-rich arc magmas. High-SiO_2_-rich magmas with increasing Dy/Yb are rare in the global dataset and might be related to fractionation of garnet (e.g. Davidson et al. 2007) and/or zircon

One of the interesting aspects of our data is the fairly systematic decrease of bulk rock Nb/Ta and Dy/Yb with increasing SiO_2_. Despite significant scatter in the data both the calk-alkaline and the high-K_2_O-Paine series display a general decrease of Nb/Ta with increasing SiO_2_ (Fig. 6). Such a weak negative correlation of SiO_2_ and Nb/Ta has also been observed in modern island arc settings in so-called adakites from the Philippines (e.g. Prouteau et al. 2000). The weak negative correlation of Nb/Ta ratios with SiO_2_ can also be reproduced by a compilation of Nb/Ta ratios from global arc magmas from the Georoc database (http://georoc.mpch-mainz.gwdg.de/georoc/), as illustrated in Fig. 10a.

This general decrease is surprising as experimentally derived Nb–Ta partitioning data of Fe–Ti oxides (magnetite, ilmenite, rutile and titanite) all show a strong preference of Ta over Nb (e.g. Green and Pearson 1986; Prowatke and Klemme 2005; Xiong et al. 2011; Nielsen and Beard 2000; Schmidt et al. 2004; Sievwright et al. 2017), independent of whether Nb and Ta are incompatible (titanomagnetite) or compatible (titanite, rutile, ilmenite). One idea is that such low Nb/Ta ratios of evolved magmas are inherited from sources with a subchondritic Nb/Ta ratio, e.g. subducted rutile-bearing eclogite (e.g. Rudnick et al. 2000). However, this hypothesis makes the assumption that these ratios are nearly unmodified since the dehydration and/or melting process in the subducted plate, an unlikely scenario.

If Fe–Ti oxides were to control the evolution of the Nb/Ta ratio of derivative magmas during crystallization, one would expect to see an increase of the Nb/Ta ratio with increasing SiO_2_, which, overall, is not the case (Fig. 10a). An alternative is that Ti (and Nb–Ta) bearing silicates control the evolution of the Nb/Ta ratio, most notably amphibole and biotite. Although the importance of amphibole for chemical differentiation of high-silica igneous rocks is well known (e.g. Cawthorn et al. 1973; Cawthorn and O’Hara 1976; Davidson et al. 2007), there are few studies that explore amphibole-sensitive trace element ratios of derivative silica-rich rocks to test the importance of amphibole for igneous differentiation. Our data show that both the calc-alkaline and the high-K_2_O-Paine series follow trajectories of decreasing TiO_2_ with increasing SiO_2_, albeit at different absolute concentrations (Figure S6 in the electronic appendix), favouring an important role for amphibole fractionation (e.g. Jagoutz et al. 2011). While Nb and Ta are moderately incompatible in amphibole in calc-alkaline compositions (Nandedkar et al. 2016; Li et al. 2017), Nb and Ta are compatible in biotite (e.g. Nash and Crecraft 1985; Acosta-Vigil et al. 2010), and partition coefficients ^Min/L^D_Nb_/^Min/L^D_Ta_ are generally > 1, unlike Fe–Ti oxides. We used the fractional crystallization experiments of Nandedkar et al. (2014, 2016) to develop a model of crystal fractionation relevant for intermediate to silicic magmas in calc-alkaline systems, and its control on trace element ratios. Since the experimental data were specifically designed to simulate the liquid line of descent of calc-alkaline magmas, we used the phase proportions and compositions of the major crystallizing phases and amphibole trace element partition coefficients as a function of SiO_2_, to develop a model evolution of SiO_2_ and Nb/Ta, assuming that amphibole and biotite are the major phase controlling this ratio (low Ti–magnetite was neglected for the Nb/Ta calculations, as partition coefficients for Nb and Ta are generally lower than 0.1). We first calculated by mass balance the evolution of liquid SiO_2_ by fractionating amphibole, plagioclase, apatite and Fe–Ti oxides in steps of 3% crystallization from 52 to 75 wt% SiO_2_, in proportions as determined from fractional crystallization experiments (Nandedkar et al. 2014). We then used the ^Amph/L^D_Nb_/^Amph/L^D_Ta_ from Nandedkar et al. (2016) and the ^Bt/L^D_Nb_/^Bt/L^D_Ta_ ratios from Stepanov and Hermann (2013), and Nash and Crecraft (1985) for simulating the evolution of the Nb/Ta ratio (Fig. 10c). The background model is an amphibole-only model similar to Li et al. (2017) while in the second and third model biotite in various proportions (Fig. 10) joins the fractionating assemblage at ~ 55–70 wt% SiO_2_. Saturation in phlogopite/biotite during fractional crystallization is strongly dependent on the initial K_2_O content of the liquid (e.g. Edgar and Arima 1983; Righter and Carmichael 1996; Sisson et al. 2005) and, therefore, we have chosen three different SiO_2_ contents to illustrate the effects of biotite fractionation on the Nb/Ta ratio of derivative liquids. Model parameters, partition coefficients, and all calculations can be found in Table S7 in the electronic appendix. The ^Amph/L^D_Nb_/^Amph/L^D_Ta_ for calc-alkaline systems varies between about 0.9 at ~ 50 wt% SiO_2_ and 1.4 at 65 wt% SiO_2_ (Nandedkar et al. 2016; Li et al. 2017), while ^Bt/L^ D_Nb_ / ^Bt/L^ D_Ta_ ratios vary between 1.8 and 4.8 (Stepanov and Hermann 2013; Nash and Crecraft 1985) for dacitic to rhyolitic liquids. We adapted the ^Amph/L^D_Nb_/^Amph/L^D_Ta_ for our models as a function of SiO_2_ according to the experimentally derived values from Nandedkar et al. (2016) for variably evolved magmas. Modelling results show that amphibole fractionation only produces Nb/Ta ratios that decrease by less than 2 units (in our model from 15 to 13). Modelling using the phase proportions of fractional crystallization experiments without biotite indicate that the Nb/Ta ratio cannot be lowered sufficiently to explain the rather low Nb/Ta of many samples exceeding 65 wt% SiO_2_. The models combining amphibole and biotite fractionation show a pronounced decrease of Nb/Ta ratios to below 10 and provide a better fit for many dacitic to rhyolitic liquids (Fig. 10c). Such a decrease can also be observed within the K-rich arc rocks from the Dariv Range in Mongolia, where mass balances used to simulate the liquid lines of descent indicates a decrease in the Nb/Ta ratio of derivative silica-enriched compositions (Bucholz et al. 2014). The remarkable decrease of average Nb/Ta of global arc magmas with SiO_2_ > 68 wt% is consistent with an important role of biotite fractionation for rhyodacite and rhyolite. Nb/Ta ratios between 10 and 6 in the Cerra Galan ignimbrites from Central Chile (Kay et al. 2005) can be explained by an important role of biotite, and melting of rutile-bearing residues or low degree of amphibolite melting are not required. For the case of the siliceous rocks from the Torres del Paine area, we conclude that Nb/Ta fractionation is controlled by amphibole and mainly by biotite, and that fractionating Fe–Ti oxides are not required to explain the data. Liquid lines of descent models of biotite-dominated high-K (shoshonitic or lamproitic) arc magmas also indicate decreasing Nb/Ta at low SiO_2_ (e.g. Bucholz et al. 2014) while silica undersaturated alkaline magmas dominated by Fe–Ti oxide fractionation should develop increasing Nb/Ta with increasing SiO_2_.

The models presented here have significant implications for the control of some trace element ratios of crustal igneous rocks. It has been proposed that the Nb/Ta ratio of continental crust is controlled by amphibolite melting or eclogite melting in subduction zones (e.g. Foley et al. 2002). While these processes might be important to regulate the initial Nb/Ta ratio of fractionating basaltic magmas, which vary widely, the results presented here appear as a natural consequence of simple fractionation of ferromagnesian silicates such as biotite ± amphibole, consistent with a global decrease of Nb/Ta ratios for more SiO_2_-rich volcanic and plutonic rocks.

A similar effect was shown for partial melting of metapelitic crustal rocks. Melting in the presence of residual biotite generated high Nb/Ta restites whose superchondritic Nb/Ta was locked in by the formation of peritectic rutile, in which Nb is highly compatible (Stepanov and Hermann 2013). Stepanov et al. (2014) showed that this can produce low Nb/Ta granites if partial melting does not occur at too high temperatures. The effect is particularly pronounced at low degrees of partial melting. Thus, partial melting of lower continental crust can also generate low Nb/Ta magmas at high SiO_2_ contents. However, this process represents reworking of existing continental crust, whereas our model imparts significantly subchondritic Nb/Ta during the fractionation of newly formed continental crust as it is produced in arcs. Moreover, we provide the first evidence that accumulation of amphibole and biotite during fractionation of arc magmas in itself is expected to generate low-Nb/Ta upper crustal granitic magmas, thus imparting a low Nb/Ta to the upper crust. As arcs represent the main location of generation of new continental crust, this is expected to be a volumetrically significant process at the crustal level.

We applied similar models to illustrate the evolution of the Dy/Yb ratios of fractionating calc-alkaline magmas. Compiled primary arc melts with ~ 50 wt% SiO_2_ have Dy/Yb ratios between 1.5 and 3 (Kelemen et al. 2003) consistent with our Dy/Yb ratios at ~ 50 wt% SiO_2_. All the modally important fractionating phases in our model (amphibole, plagioclase, and apatite) have ^Min/L^ D_Dy_ / ^Min/L^ D_Yb_ > 1, with amphibole ranging between 1.11 and 1.27 (Nandedkar et al. 2016) and apatite ranging from 2.2 to 2.9 (Prowatke and Klemme 2006), amplifying the effect of amphibole in decreasing Dy/Yb with increasing SiO_2_. Although plagioclase is an important fractionating phase, middle to heavy REE are incompatible in plagioclase and its influence on the Dy/Yb ratio of derivative liquids is minor. Minerals with ^Min/L^D_Dy_/^Min/L^D_Yb_ < 1 are garnet, opx and for highly evolved compositions, zircon. If one of these phases is volumetrically important the Dy/Yb ratio will follow a different trajectory with increasing SiO_2_. Our fractional crystallization modelling involving phase proportions and compositions of Nandedkar et al. (2014) shows that the evolution of the Dy/Yb ratio steadily decreases, amplified if apatite joins the crystallizing assemblage at ~ 69 wt% SiO_2_ (Fig. 10d). A tiny amount of zircon, however, led to a significant increase in the Dy/Yb ratio. The simple models presented here indicate that the Dy/Yb ratio of the rocks from the Torres del Paine area are controlled by amphibole ± plagioclase and apatite. It is remarkable that the evolution of the two fractionation series of the Paine area is essentially parallel (Fig. 10c), albeit with a different initial Dy/Yb ratio, which might be related to different degrees of melting in the mantle wedge. This is consistent with findings of Davidson et al. (2007) that the evolution of Dy/Yb ratios can only be identified for a single magmatic series. This indicates that the two fractionation series are mainly controlled by the same mineral phase equilibria.

## Conclusions

The Torres del Paine area provides an episodic record of alkaline rear arc and calc-alkaline arc magmatism demonstrating lateral variability of arc magmatism in the Southern Patagonian batholith over the last 30 Ma. Our new U–Pb data on zircon indicate that the growth of the Southern Patagonian batholith is primarily dominated by igneous fractionation and that recycling of pre-existing material as recorded by inherited zircons is not fundamental for the composition of derivative silica-rich magmas in Southern Patagonia. Igneous rocks that crystallized between about 17–16 and 12.5 Ma show trace element ratios (Th/Nb, Th/La) with an important subduction related component, which is absent in ~ 29–30 Ma old and very recent alkaline rocks. This increased subduction component is mainly recorded in the 17–16 my calc-alkaline series (e.g. elevated Th/Nb, and La/Nb), probably related to a period of fast convergence of the Nazca and South American plate during the Miocene. During this period of fast convergence, eastward migration of the magmatic arc occurred by ~ 65 km in the Torres del Paine area and ~ 100 km in the Chalten area, making subduction erosion a likely process to contribute to the chemistry of subduction-related igneous rocks between 17 and 16 Ma (see also Ramírez de Arellano et al. 2012). Simultaneous arc migration and forearc loss has also been shown for the south–central Andes (e.g. Kay et al. 2005).

Simple model calculations based on fractional crystallization experiments in calc-alkaline systems document that trace element ratios such as Dy/Yb in evolved silica-rich magmas may be controlled by amphibole fractionation, while Nb/Ta is essentially controlled by biotite ± amphibole fractionation in the middle to lower crust. Other processes related to reworking of existing continental crust during partial melting in the presence of biotite (Stepanov and Hermann 2013), amphibolite melting in subduction zones (e.g. Foley et al. 2002), or more complex processes such as diffusive fractionation of Nb/Ta in rutile (e.g. Marschall et al. 2013) are probably not required to derive low Nb/Ta ratios of upper crustal igneous rocks.

## Electronic supplementary material

Below is the link to the electronic supplementary material.

Supplementary material 1 (PDF 3397 KB)

S1: Bulk rock major and trace element analysis. S2-S6: U–Pb data for primary and secondary standards and unknowns. S7: Fractionation model calculations and references (XLSX 220 KB)
