# Supplementary material for: Source and fractionation controls on subduction-related plutons and dike swarms in southern Patagonia (Torres del Paine area) and the low Nb/Ta of upper crustal igneous rocks
Source: Contrib Mineral Petrol. 2018 Apr 19;173(5):38. doi: 10.1007/s00410-018-1467-0 (PMC5907628; doi:10.1007/s00410-018-1467-0)
Supplement: Supplementary file 1 — Supplementary material 1 (PDF 3397 KB) [file 410_2018_1467_MOESM1_ESM.pdf]

Supplementary online resources for

Source and fractionation controls on subduction-related plutons and dike swarms in Southern Patagonia (Torres del Paine area)

Müntener O[1]*, Ewing TA[1,2], Baumgartner LP[1], Manzini M[1], Roux T[1], Pellaud P[1] and Allemann L[1]

[1]Institute of Earth Sciences, University of Lausanne, CH-1015 Lausanne, Switzerland
[2]Present address: Institute of Geological Sciences, University of Bern, CH-3012 Bern, Switzerland

*Corresponding author: *othmar.muntener@unil.ch*, *+41 21 692 43 47*

Contributions to Mineralogy and Petrology (2018)

S1 Detailed description of zircon characteristics and U–Pb data

Zircons from all samples are euhedral and appear clean and transparent in transmitted light, with the exception of radiation-damaged zircons from 11-TPM-73A that are dark and opaque (see below). In eight of the twelve samples there is no evidence in cathodoluminescense for more than one generation of zircon growth (Fig. 6, main text). In CL, these zircons display well-developed oscillatory zoning (sample LG-026A); oscillatory zoning or rather homogeneous CL emission (11-TPM-73A, 11-TPM-85, 11-TPM-32A); or faint panelled zoning or occasionally oscillatory zoning (PP-36, 11-TPM-61A, 07-JL-204) (Fig. 6). The four remaining samples have more complex CL characteristics, which are discussed in the main text.

Intrusive bodies

Zircons from the monzogabbro **11-TPM-73A** contain occasional inclusions. In transmitted light many zircons are extremely dark and appear strongly radiation damaged, this often being associated with unusual patchy CL emission. Most zircons are very dark in CL. Of thirteen analyses, four are excluded as discordant (Fig. S3). Two of the remaining analyses have distinctly younger ages, one of which is discordant. These zircons have extraordinarily high Th contents (∼18000 ppm, at least twice as much as for other zircons). Their young dates are therefore attributed to Pb loss due to radiation damage. The remaining seven concordant analyses still have very scattered ^{206}Pb/^{238}U dates, with an MSWD of 11 (Fig. 7), but no justification was found for excluding further analyses. All zircons from this sample analysed for trace elements have rather high Th and U contents (900–18000 ppm and 700–7000 ppm, respectively; Manzini, 2012). They have high Th/U of 0.4–13. The high concentrations of U and particularly Th mean that these zircons may have suffered radiation damage and associated Pb loss, which may have contributed to the scatter in dates observed for this sample. The age of this sample is constrained only approximately to **∼29–30 Ma**, defined by seven analyses that are concordant but show excess scatter (MSWD = 11). This age is nonetheless clearly distinguished from the age of all other samples from this study, and is in line with a K–Ar age of a similar gabbro (29.4 ± 0.8 Ma; Altenberger, 2003).
Zircons from the calc-alkaline Oldivado intrusion (sample **13-OLV-1**, see Fig. 1) contain abundant small inclusions. Of seventeen zircons analyses, one gave a strongly discordant date that is distinctly older than the main population in terms of both ^{206}Pb/^{238}U and ^{207}Pb/^{206}Pb, but does not fall on the trend towards ^{207}Pb defined by other analyses (Fig. 8a). Post-analysis inspection revealed that this spot was partly on a central core domain with extremely bright CL emission, but also partly overlapped the surrounding oscillatory-zoned rim that is much dimmer in CL (Fig. 6a). The CL-bright central domain is interpreted as an inherited core, and this analysis as a mixed core–rim age. This analysis does not have significantly higher ^{208}Pb than other analyses from the same sample, indicating that there is little or no common Pb contribution. Its ^{207}Pb/^{206}Pb date of 2710 ± 90 Ma is therefore taken as a minimum age for the inherited core. The strong discordance of this analysis, with a ^{206}Pb/^{238}U date of 290 ± 30 Ma, is at least in part due to mixing with the much younger rim domain, although partial Pb loss may also have affected it. Of the remaining sixteen analyses, seven were excluded as discordant (Fig. S3). The nine concordant analyses give a weighted mean ^{206}Pb/^{238}U age of **15.7 ± 0.4 Ma** with an MSWD of 1.0 (Fig. 7).
Zircons from sample **13-TP-2** (small intrusion Paso Gardner, Fig. 1) display two distinct patterns in CL: (1) typical oscillatory zoning, and (2) more blocky, less regular CL patterns sometimes resembling sector zoning. Rarely both are observed in one zircon, with the sector-type zircon appearing to discordantly replace the oscillatory-zoned zircon. However, no resolvable difference in age was found between these two types of zone. Of seventeen analyses, three are excluded as discordant (Fig. S3), leaving fourteen concordant analyses that give a weighted mean ^{206}Pb/^{238}U age of **15.9 ± 0.4 Ma** with an MSWD of 0.7 (Fig. 7).

Zircons from sample **09-PR-06** (Granite SW of Paine Grande, Fig. 1) often contain abundant inclusions, although some inclusion-free zircons are also found. In CL zircons display well-developed oscillatory zoning and sometimes areas of patchy or homogeneous CL emission. A few zircons have central domains that are sometimes partially resorbed or replaced (e.g. Fig. 6c). Two analyses of such domains gave significantly older dates that do not fall on the trend of discordant analyses towards ^{207}Pb (Fig. 10b), and are interpreted as dating inherited cores. One gave a concordant date with a ^{206}Pb/^{238}U age of 565 ± 5 Ma (Fig. 10b). This analysis was on a central domain of a zircon with homogenous medium CL emission, and an irregular contact with the surrounding oscillatory-zoned rim (Fig. 6b). The other inherited date is subconcordant with a ^{207}Pb/^{206}Pb date of 2720 ± 10 Ma and a ^{206}Pb/^{238}U date of 2520 ± 20 Ma (Fig. 10b). The small difference in age is interpreted to result from a low degree of Pb loss, and the ^{207}Pb/^{206}Pb age of 2720 ± 10 Ma best records the age of this inherited domain. This analysis is on a partially resorbed oscillatory-zoned central domain with a discordant relationship to the surrounding oscillatory zoned rim and patchy replacement by CL-homogeneous zircon (Fig. 6c). Of the remaining 31 analyses, five were excluded as discordant (Fig. S3). Two analyses of very inclusion-rich zircons gave distinctly younger ages that are also excluded (Fig. 7), resulting in a clear improvement in the MSWD from 2.2 to 1.3. The remaining 24 concordant analyses give a weighted mean ^{206}Pb/^{238}U age of **16.1 ± 0. 2 Ma**, with an MSWD of 1.3.

Zircons from sample **07-JL-204** (SE of Pta Bariloche, Fig. 1) sometimes contain a small number of inclusions. All thirteen analyses are concordant (Fig. 8), and give a weighted mean ^{206}Pb/^{238}U age of **16.5 ± 0.2 Ma** with an MSWD of 1.4.

Many zircons from sample **PP-36** (granite SE of Pta Bariloche, Fig. 1) contain abundant inclusions. Of thirty-one analyses, six discordant analyses were excluded (Fig. S3). The remaining twenty-five analyses are concordant (Fig. 8) and all agree within error to give a weighted mean ^{206}Pb/^{238}U age of **16.4 ± 0.2 Ma** with an MSWD of 1.5.

Zircons from sample **11-TPM-61A** only rarely contain inclusions. Thirty-seven zircon analyses all gave concordant dates that define a single population with a weighted mean age of **16.2 ± 0.2 Ma** and an MSWD of 1.3 (Fig. 8).

Most zircons from **11-TPM-94** (a small monzodiorite intrusion at the entrance of Val Bader) contain abundant inclusions. Of twenty-one analyses, eight are excluded as discordant (Fig. S3). One analysis is concordant but significantly older with a ^{206}Pb/^{238}U date of 90 2 Ma (Fig. 10c). In this study, unintended sampling of inclusions almost universally produced discordant dates defining a single trend towards ^{207}Pb. This analysis does not show this behaviour (Fig. 10c) and samples a central domain that could represent an inherited core, although from CL alone this is not unequivocal (Fig. 6d). This analysis is interpreted as dating an inherited zircon with an age of 90 ± 2 Ma. Of the remaining twelve concordant analyses, one is a distinct outlier in age (Fig. 8), and its exclusion improves the MSWD of the ^{206}Pb/^{238}U dates from 1.8 to 1.2. This analysis is on a zircon with abundant inclusions and we infer that its slightly older date reflects sampling of sub-surface inclusion(s). Excluding this analysis, the remaining eleven concordant analyses have a weighted mean age of **12.4 ± 0.2 Ma**, with an MSWD of 1.2.

Dikes

Zircons from sample **LG-026A** mostly contain abundant inclusions, and some are also heavily cracked. Thirty-six zircons were analysed, of which fifteen were excluded as discordant (Fig. S3). Of the twenty-one concordant analyses, one is a distinct outlier in ^{206}Pb/^{238}U age (Fig. 9), and probably was contaminated by a sub-surface inclusion. This outlier clearly does not belong to the main population and is excluded from the sample age calculation, noting that its inclusion or exclusion makes negligible difference to the age obtained. The remaining 20 analyses give a weighted mean ^{206}Pb/^{238}U age of **12.3 ± 0.2 Ma** with an MSWD of 1.7.

Zircons from **11-TPM-85** contain abundant inclusions. Twelve analyses were all concordant and give a weighted mean ^{206}Pb/^{238}U age of **12.3 ± 0.2 Ma** with an MSWD of 1.3 (Fig. 9).
Zircons from **11-TPM-32A** contain abundant inclusions. Of fifteen analyses, three on inclusion-rich zircons were excluded as discordant or very imprecise (Fig S3). One analysis distinctly younger in ^{206}Pb/^{238}U age, also on an inclusion-rich zircon, was excluded (Fig. 9), improving the MSWD from 3.3 to 1.5. The remaining eleven analyses give a weighted mean age of **12.8 ± 0.2 Ma**, with an MSWD of 1.5.

S2 Determination of ages from analyses uncorrected for common Pb

Given the difficulty in accurately common Pb correcting individual ICPMS analyses, there are two possible approaches to obtain sample ages that are not biased by common Pb. The first is to use a linear regression through all analyses on the Tera-Wasserburg diagram. This approach assumes that zircons all have the same crystallisation age and contain variable proportions of common Pb, but that this common Pb always has the same isotopic composition. In this case, the lower intercept of the regression with the concordia gives the U–Pb age of the zircons, while the upper intercept gives the ^{207}Pb/^{206}Pb composition of the common Pb. The second alternative is to exclude all analyses containing any common Pb, as identified by their being discordant outside error, and calculate a weighted mean ^{206}Pb/^{238}U age of the remaining (common Pb-free) analyses. We applied both methods to all of our samples, except for two that only had concordant analyses (precluding a useful regression). In all cases lower intercept ages from the regression method were indistinguishable from weighted mean ages of concordant analyses. We take the weighted mean ^{206}Pb/^{238}U age of common Pb-free (concordant) analyses as our preferred sample age. Common Pb measured on zircon may often come from ‘foreign’ sources (e.g. introduced into cracks during sample preparation, from inclusions of common Pb rich phases such as feldspar). Indeed, in many cases in this study, high common Pb analyses were associated with domains that were cracked or damaged, which would favour the incorporation of external Pb into the zircon, and not only at the surface. For this reason we regard the analyses free of common Pb as the most reliable determination of the crystallisation age of the zircon.

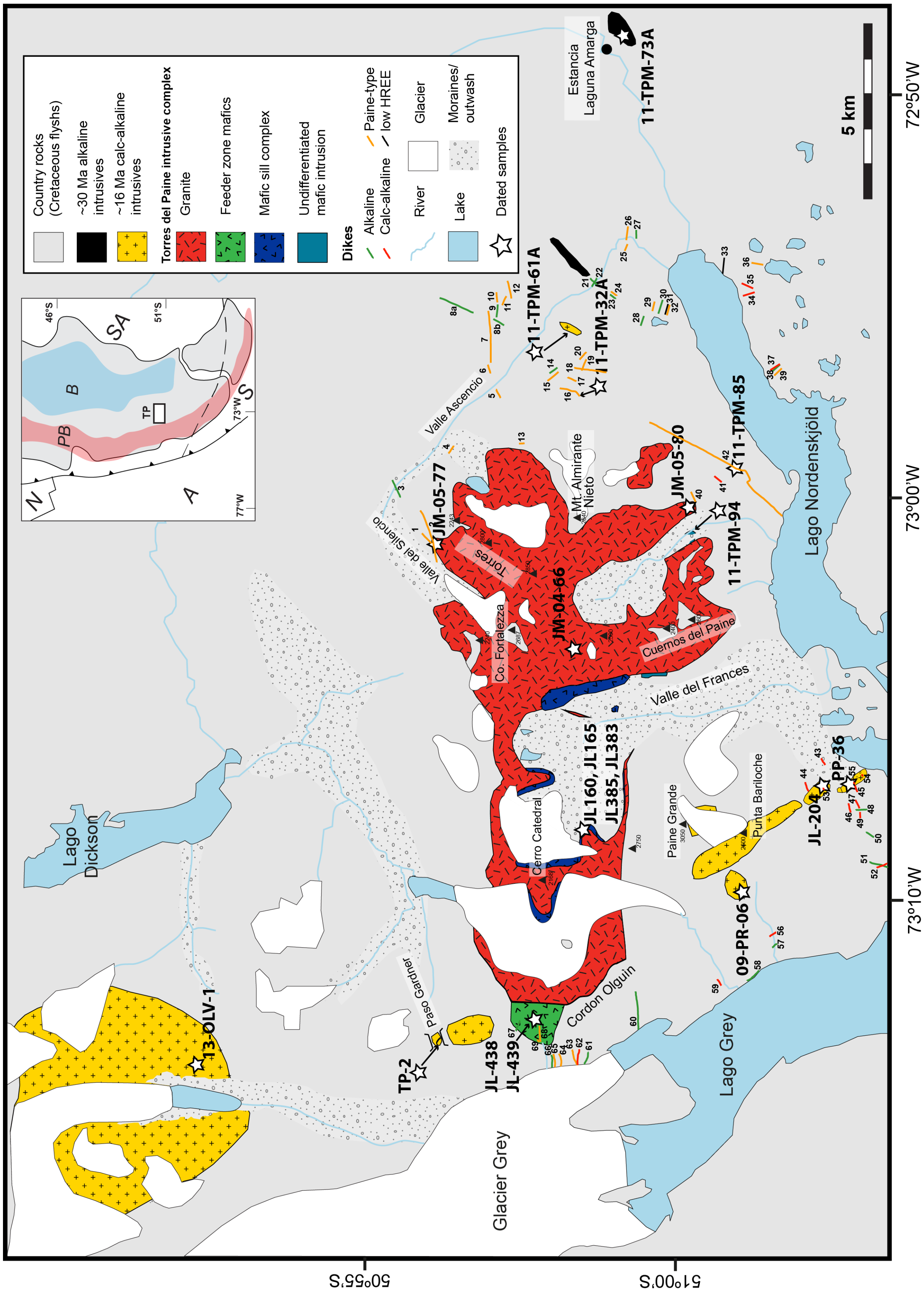

Figure S1: Simplified geological map of the Torres del Paine area with location of the different magmatic rocks, plutonics and dikes (after Michel et al., 2008; Leuthold et al., 2012). Dated samples are given with full sample number. Dikes analysed for chemistry are annotated with arbitrary numbers from 1 to 67. These numbers appear in Table S1 in the column "Number on map", which can thus be used to find the sample name of a given dike. Dikes within the felsic and mafic rocks of the Torres del Paine are not shown.

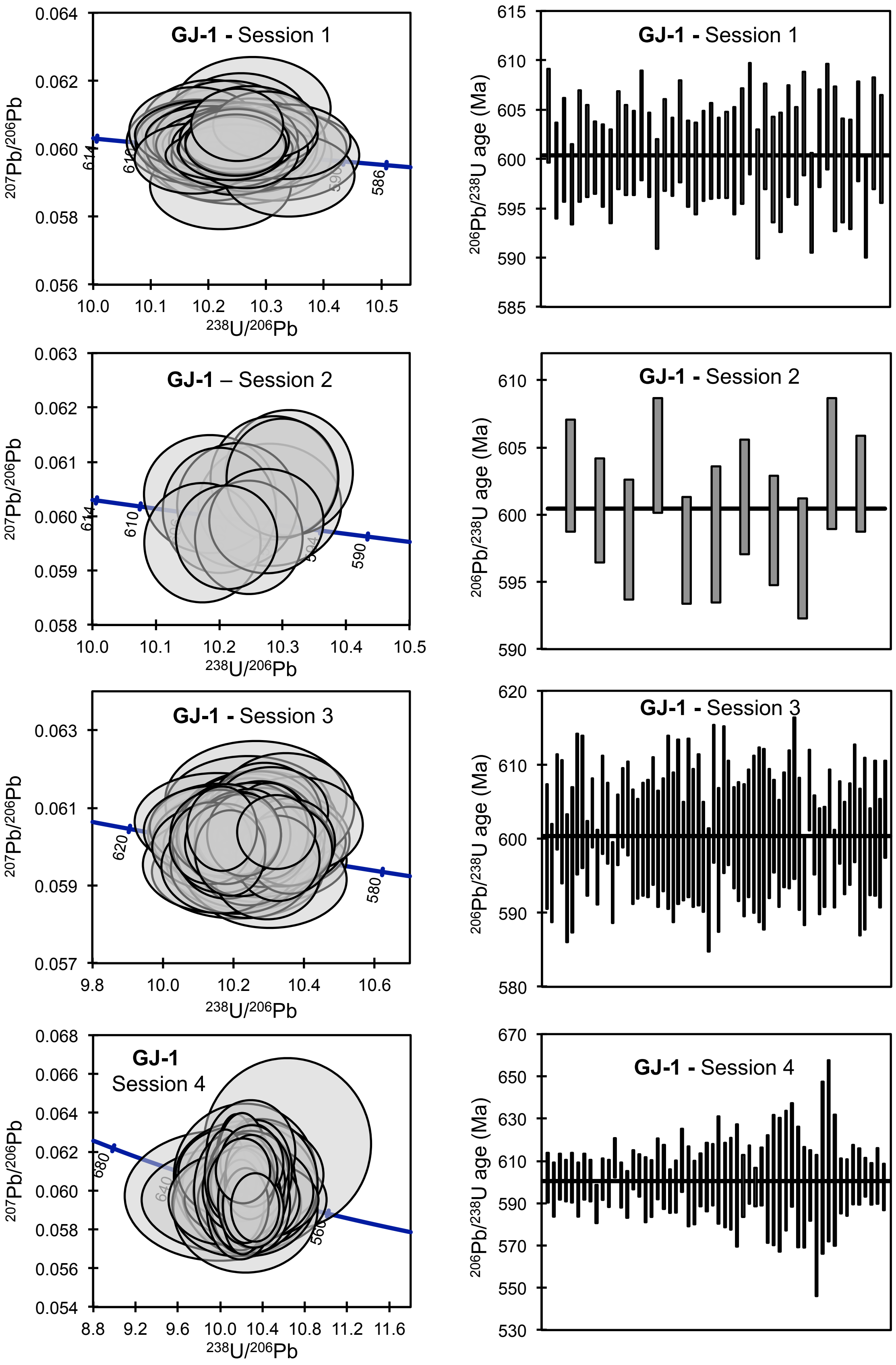

Figure S2: U–Pb data for the primary standard, GJ-1 zircon, in four sessions, plotted on Tera-Wasserburg concordia diagrams (left) and $^{206}Pb/^{238}U$ age plots (right), the latter with weighted mean shown as a black horizontal line. No analyses were excluded.

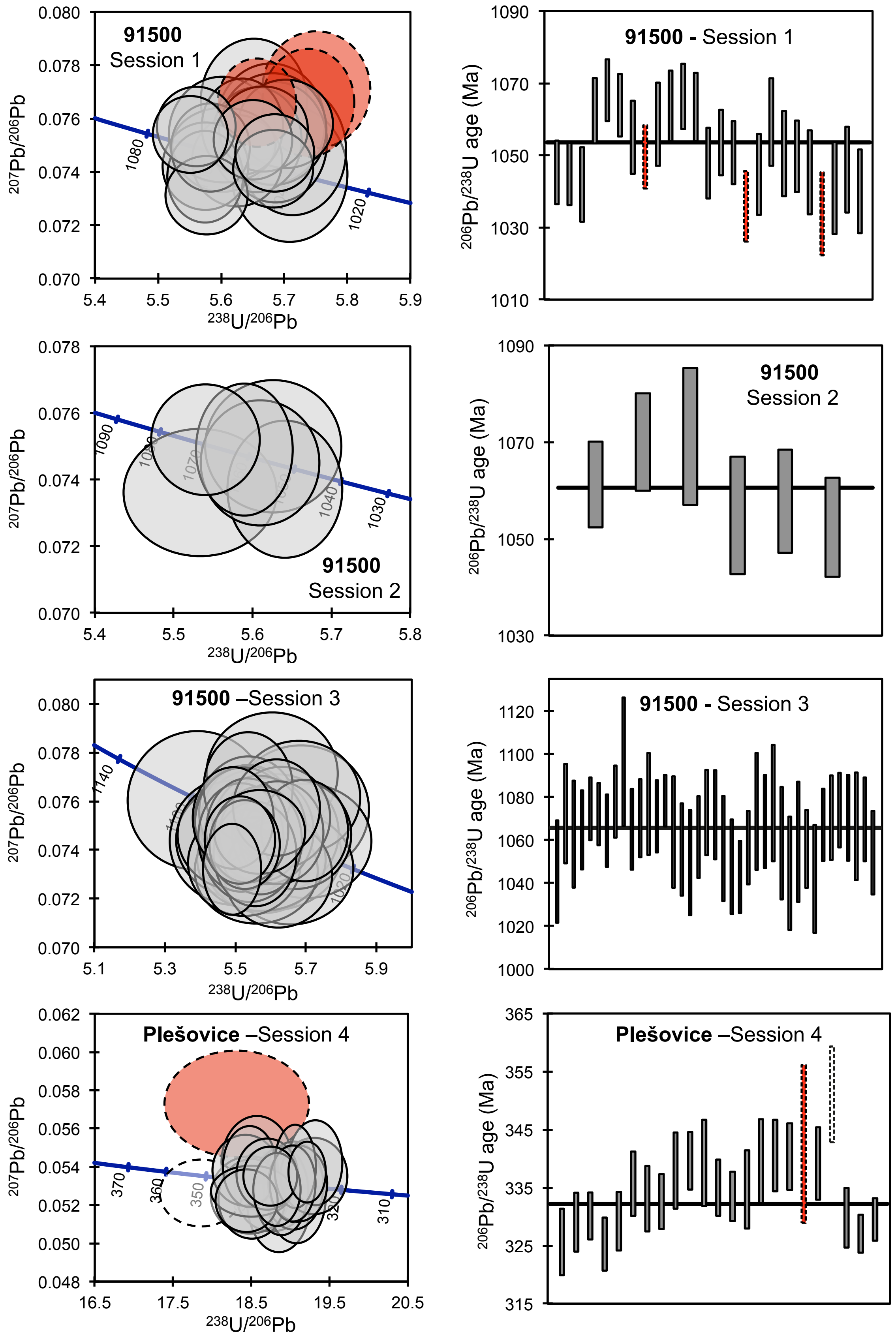

Figure S3: U–Pb data for natural zircons (91500 and Plešovice) analysed as secondary standards in four sessions, plotted on Tera-Wasserburg concordia diagrams (left) and ^{206}Pb/^{238}U age plots (right), the latter with the weighted mean shown as a black horizontal line. Analyses excluded for discordance (red fill) or being distinct outliers in ^{206}Pb/^{238}U age (white fill) are shown with a dashed outline. Nominal ages are 1065 Ma for 91500 (Wiedenbeck et al., 1995) and 337.13 ± 0.37 Ma for Plešovice (Slama et al., 2008).

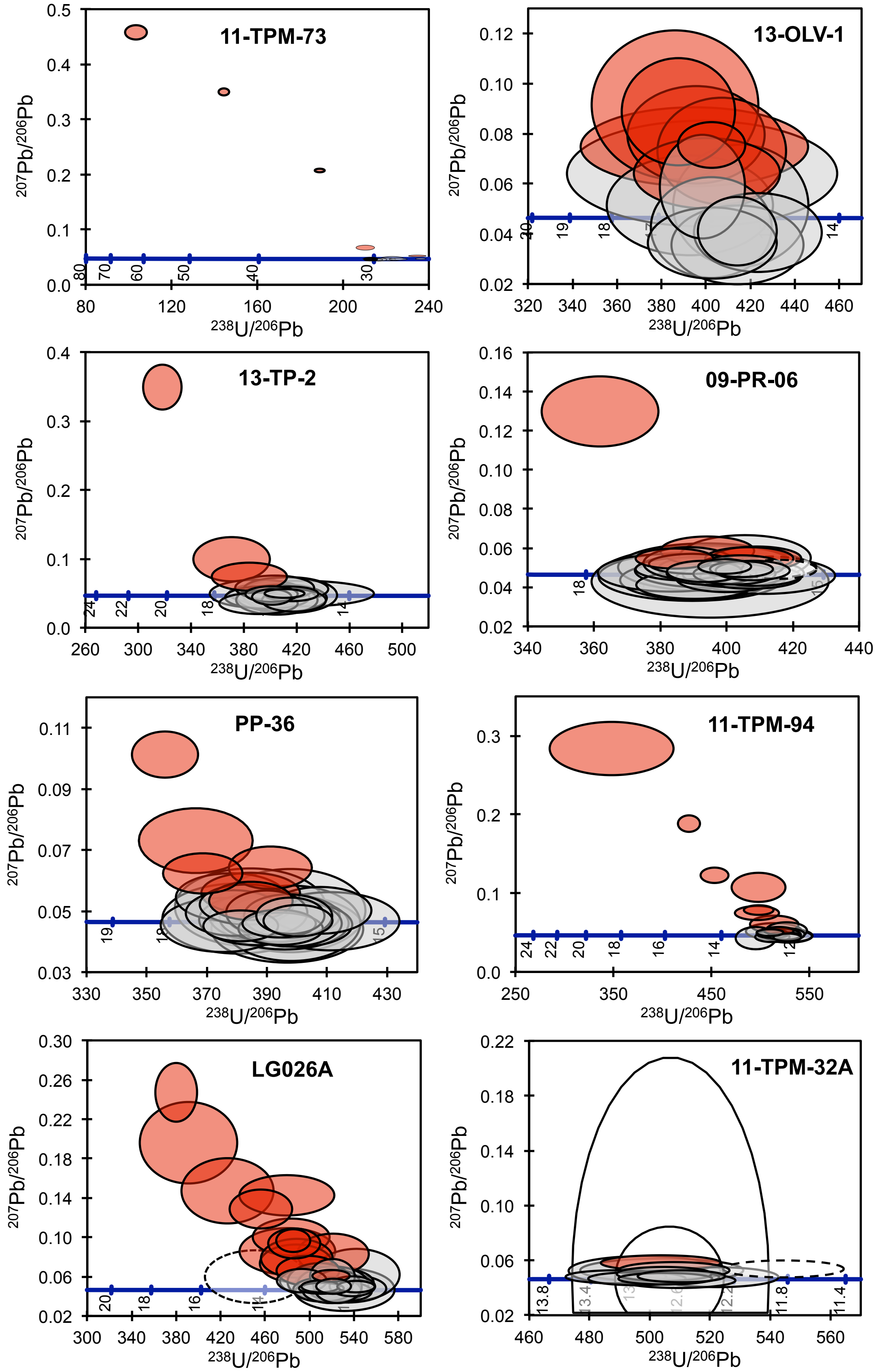

Figure S4: Tera-Wasserburg concordia diagrams for samples of intrusive bodies and dikes from the Torres del Paine region, including those analyses excluded from the sample age calculation because they were discordant (red ellipses), very imprecise (white ellipse with solid border), or distinct outliers (white ellipse dashed border). Gray ellipses are analyses included in the age calculation. Analyses of inherited core domains are not plotted. Samples 11-TPM-61A, 11-TPM-85 and 09-JL-204 are not shown as all analyses were used in the age calculation for these samples.

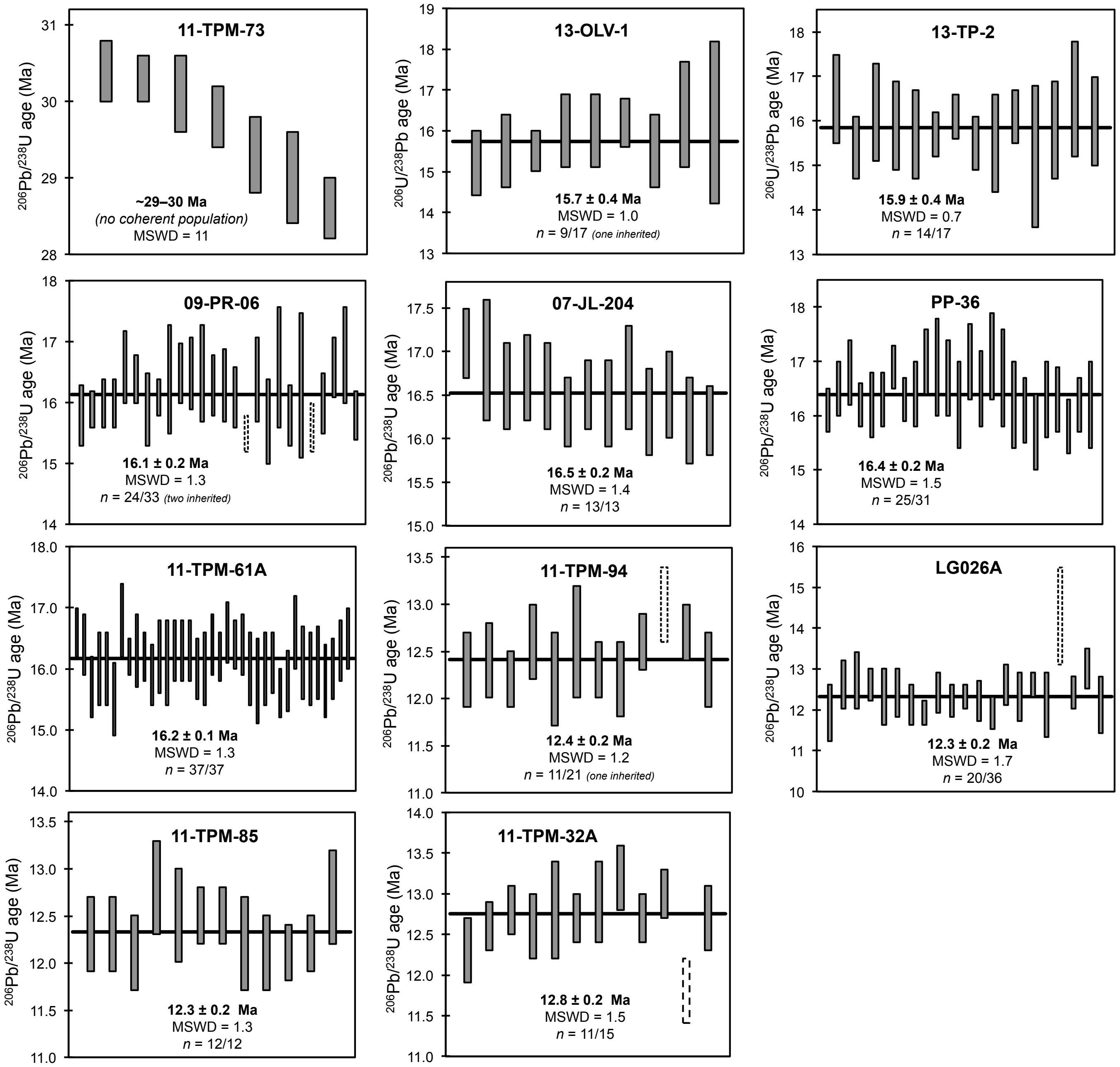

Figure S5: ^{206}Pb/^{238}U dates used to calculate sample U–Pb ages for eight intrusive bodies and three dikes from the Torres del Paine region. Weighted average ^{206}Pb/^{238}U age is shown as a horizontal line and quoted as text. Analyses excluded as outliers in age are shown by a dashed line. All other excluded analyses (e.g. for high common Pb, inclusions; see Fig. S4) are not plotted. The number of analyses used to calculate the sample age as a fraction of the total number of analyses is given as n.

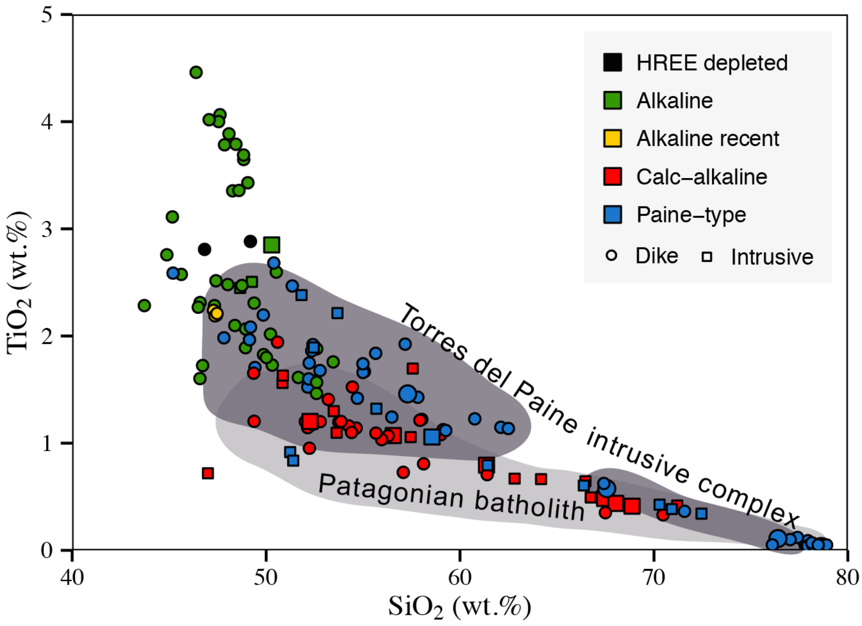

Figure S6: Bulk rock TiO_2 versus SiO_2 (anhydrous values) for magmatic rocks from the Torres del Paine region. Larger symbol size indicates samples that were dated by U–Pb on zircons. Field for the Torres del Paine intrusive complex compiled from Michael (1984) and Leuthold et al. (2013). Patagonian batholith field from data of Herve et al. (2007), ranging from late Jurassic to the Neogene.

Bibliography

Altenberger (2003). Tectonic controls and Cenozoic magmatism at the Torres del Tectonic controls and Cenozoic magmatism at the Torres del Paine, southern Andes (Chile, 51 °10'S). *Revista Geológica de Chile*, **30**(1), 65–81.

Herve, F., Pankhurst, R. J., Fanning, C. M., Caldéron, M., and Yaxley, G. M. (2007). The South Patagonian batholith: 150 my of granite magmatism on a plate margin. *Lithos*, **97**(3-4), 373–394.

Leuthold, J., Müntener, O., Baumgartner, L. P., Putlitz, B., and Chiaradia, M. (2013). A Detailed Geochemical Study of a Shallow Arc-related Laccolith; the Torres del Paine Mafic Complex (Patagonia). *Journal of Petrology*, **54**(2), 273–303.

Leuthold, J., Müntener, O., Baumgartner, L. P., Putlitz, B., Ovtcharova, M., and Schaltegger, U. (2012). Time resolved construction of a bimodal laccolith (Torres del Paine, Patagonia). *Earth and Planetary Science Letters*, **325–326**, 85–92.

Manzini, M. (2012). Géochimie et géochronologie des roches ignées du SE du parc national de Torres del Paine, Chili. Master's thesis, University of Lausanne, 116p.

Michael, P. J. (1984). Chemical differentiation of the Cordillera Paine granite (southern Chile) by in situ fractional crystallization. *Contributions to Mineralogy and Petrology*, **87**, 179–195.

Michel, J., Baumgartner, L., Putlitz, B., Schaltegger, U., and Ovtcharova, M. (2008). Incremental growth of the Patagonian Torres del Paine laccolith over 90 k.y. *Geology*, **36**(6), 459–462.

Slama, J., Kosler, J., Condon, D. J., Crowley, J. L., Gerdes, A., Hanchar, J. M., Horstwood, M. S. A., Morris, G. A., Nasdala, L., Nicholas, N., Schaltegger, U., Schoene, B., Tubrett, M. N., and Whitehouse, M. J. (2008). Plešovice zircon – A new natural reference material for U–Pb and Hf isotopic microanalysis. *Chemical Geology*, **249**, 1–35.

Wiedenbeck, M., Allé, P., Corfu, F., Griffin, W. L., Meier, M., Oberli, F., Quadt, A., Roddick, J. C., and Spiegel, W. (1995). Three natural zircon standards for U-Th-Pb, Lu-Hf, trace element and REE analyses. *Geostandards Newsletter*, **19**(1), 1–23.
